# Unveiling the physiological impact of ESCRT-dependent autophagosome closure by targeting the VPS37A ubiquitin E2 variant-like domain

**DOI:** 10.1016/j.celrep.2024.115016

**Published:** 2024-11-27

**Authors:** Kouta Hamamoto, Xinwen Liang, Ayako Ito, Matthew Lanza, Van Bui, Jiawen Zhang, David M. Opozda, Tatsuya Hattori, Longgui Chen, David Haddock, Fumiaki Imamura, Hong-Gang Wang, Yoshinori Takahashi

**Affiliations:** 1 Division of Pediatric Hematology and Oncology, Department of Pediatrics, The Pennsylvania State University College of Medicine, Hershey, PA 17033, USA; 2 Department of Pharmacology, The Pennsylvania State University College of Medicine, Hershey, PA 17033, USA; 3 Department of Comparative Medicine, The Pennsylvania State University College of Medicine, Hershey, PA 17033, USA; 4 Department of Pathology and Biochemistry, The Pennsylvania State University College of Medicine, Hershey, PA 17033, USA; 5 These authors contributed equally; 6 Lead contact

## Abstract

Macroautophagy (autophagy) involves the formation of phagophores that mature into autophagosomes. The impact of inhibiting autophagosome closure remains unclear. Here, we report the generation and analysis of mice with impaired autophagosome closure by targeting the ubiquitin E2 variant-like (UEVL) β strands of the endosomal sorting complex required for transport (ESCRT) I subunit VPS37A. The VPS37A UEVL mutation (Δ43–139) impairs bulk autophagic flux without disrupting ESCRT-I complex assembly and endosomal function. Homozygous mutant mice exhibit signs of autophagy impairment, including p62/SQSTM1 and ubiquitinated protein accumulation, neuronal dysfunction, growth retardation, antioxidant gene upregulation, and tissue abnormalities. However, about half of the mutant neonates survive to adulthood without severe liver injury. LC3 proximity proteomics reveals that the VPS37A UEVL mutation leads to active TANK-binding kinase 1 (TBK1) accumulation on phagophores, resulting in increased p62 phosphorylation and inclusion formation. These findings reveal a previously unappreciated role of LC3-conjugated phagophores in facilitating protein aggregation and sequestration, potentially alleviating proteotoxicity.

## In brief

Hamamoto et al. report that disrupting the VPS37A ubiquitin E2 variant-like domain impairs autophagosome closure, resulting in milder autophagy-defective phenotypes in mice compared to those lacking upstream autophagy-related genes. They found that LC3-conjugated phagophores facilitate TBK1-dependent p62 phosphorylation, potentially alleviating proteotoxicity by sequestering protein aggregates.

## Graphical abstract

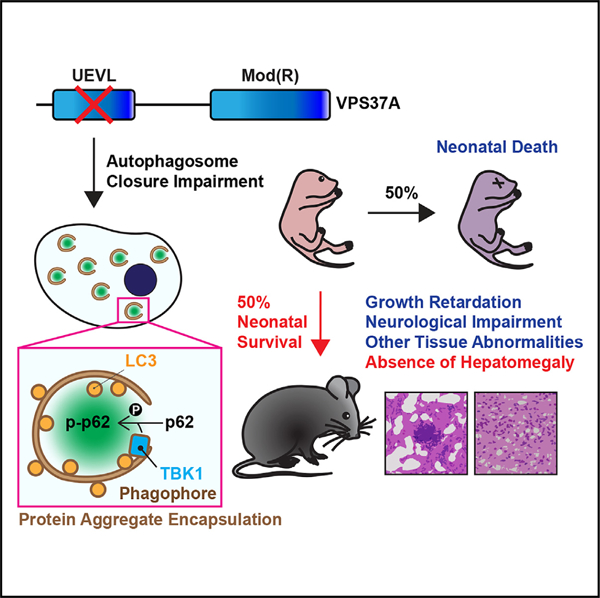

## INTRODUCTION

Macroautophagy, hereafter referred to as autophagy, is a major lysosomal degradation pathway for nutrient recycling and cellular quality control. The process of autophagy begins with the formation of a crescent-shaped membrane structure, known as the phagophore, which selectively or non-selectively sequesters cytoplasmic constituents such as proteins, macromolecules, and organelles. The formation and growth of the phagophore is coordinated by a series of autophagy-related (ATG) proteins, including the ULK1/ATG1 complex (ULK1/2, ATG13, RB1CC1/FIP200, and ATG101) for initiation, class III phosphatidylinositol 3-kinase complex I (PIK3C3/VPS34, PI3KR4/VPS15, BECN1, and ATG14) for nucleation, and the LC3/ATG8 conjugation system (ATG7, ATG3, ATG10, ATG12, ATG5, ATG16L, and MAP1LC3/GABARAP/ATG8), together with the ATG2 complex (ATG2A/B and WIPI) and ATG9A, for membrane expansion.^1–3^ Once covalently conjugated with phosphatidylethanolamine on the phagophore, LC3/GABARAP also mediates cargo recruitment through autophagy receptors such as p62/SQSTM1.^4^ Upon membrane growth, the phagophore eventually seals to form a double membrane vesicle called the autophagosome. The endosomal sorting complex required for transport (ESCRT) machinery mediates scission of the phagophore to close the membrane neck and separate the inner and outer membranes of the autophagosome. To date, components of the ESCRT-I (VPS37A, TSG101, and VPS28) and ESCRT-III (CHMP4B and CHMP2A) complexes and the AAA ATPase VPS4 have been identified as critical ESCRT factors for mammalian autophagosome closure.^5–8^ The vesicle closure step is critical for proper fusion with lysosomes to digest sequestered contents.^6,9^

The physiological importance of autophagy has been studied extensively using mice deficient in genes that are directly implicated in or function upstream of LC3 conjugation.^10^ Mice systemically lacking the core ATG genes die either during embryogenesis (RB1CC1, ATG13, BECN1, PIK3C3, and ATG9A) or shortly after birth (ULK1/2, ATG7, ATG12, ATG5, ATG16L, and ATG3). The underlying mechanisms of the variation in mortality remain unclear but may be related to their other functions besides autophagic degradation. The effects of autophagy inhibition in adult mice have also been examined using conditional gene knockout systems. Adult mice systemically depleted of ATG7 using the tamoxifen-inducible gene deletion system show liver damage, muscle wasting, adipose loss, testicular degeneration, and lethal neurodegeneration.^11^ Likewise, mice lacking ATG5 in all tissues excepts the brain also exhibit liver and other tissue damage along with growth retardation,^12^ while neuronal loss of ATG5 or ATG7 causes neurodegeneration.^13,14^ The tissue abnormalities of these autophagy-defective mice are often accompanied by the accumulation of p62 and ubiquitinated proteins. In the liver, p62 sequesters the E3 ligase KEAP1 and stabilizes the antioxidant transcription factor NRF2/NFE2L2.^15^ The deletion of p62 or NRF2 abolishes liver damage and subsequent benign adenoma formation induced by ATG5 or ATG7 loss.^15–17^ The importance of autophagy to suppress liver damage has further been demonstrated by hepatic deletion of RB1CC1.^18^ In contrast, the physiological impact of inhibiting autophagosome biogenesis downstream of LC3 conjugation is not well understood. Mice deficient in TSG101 have been shown to die *in utero*.^19^ An embryonic lethality has also been reported in mice carrying ubiquitin binding-defective UBAP1,^20^ an ESCRT-I subunit that forms a heterotetrameric complex with TSG101, VPS28, and VPS37A. However, TSG101 is a core ESCRT-I subunit involved in various membrane remodeling processes besides autophagy.^21^ Moreover, UBAP1 is an endosome-specific ESCRT-I subunit whose loss has little effect on autophagosome closure.^7,22^ Thus, it remains unclear whether the embryonic lethality observed in these mice is simply due to autophagy impairment or other defects.

The ESCRT-I subunit VPS37A, also known as hepatocellular carcinoma-related protein 1 (HCRP1), was originally discovered as a cell growth-regulatory protein frequently downregulated in hepatocellular carcinoma (HCC).^23^ We have recently demonstrated a unique role of VPS37A in directing the ESCRT machinery to the phagophores.^7^ The autophagy-specific function of VPS37A is exerted by its N-terminal ubiquitin E2 variant-like (UEVL) domain-containing region,^24^ which is absent in other VPS37 homologs. Disruption of the VPS37A N terminus impairs autophagosome closure and results in the accumulation of LC3-positive phagophores.^7,24,25^ Interestingly, unlike most ATG genes, which are rarely lost or mutated,^26–28^ the gene encoding VPS37A is frequently lost in various types of solid cancers due to chromosome 8p deletion,^29,30^ correlating with worse survival outcomes.^31–40^ Moreover, it has been shown that LC3-positive membranes have signaling roles in cell fate besides cargo sequestration.^41–53^ To understand the physiological consequences of autophagosome closure inhibition and phagophore accumulation, we generated mice with systemic deletion of the VPS37A UEVL region (Δ43–139). The mutation successfully impaired autophagosome closure without affecting ESCRT-I complex formation and stability and systemically accumulated LC3-II and p62 in mice. Moreover, the VPS37A mutant mice exhibited phenotypes characteristic of autophagy impairment, such as neonatal lethality, growth retardation, neuronal dysfunction, and tissue abnormalities. However, several phenotypes manifested by the mutant mice, specifically neonate mortality and liver damage, were found to be less severe than those reported in ATG-deficient mice.^10^ Interestingly, despite similar levels of p62 accumulation, we found that the levels of p62 phosphorylation and aggregation were much higher in autophagosome closure-defective VPS37A mutant or knockout cells than in LC3 conjugation-defective ATG7 knockout cells. Through proximity proteomics of phagophore-conjugated LC3, we identified the IKK-related kinase TBK1 as the major kinase responsible for the enhancement of p62 phosphorylation and inclusion formation by autophagosome closure inhibition. Of note, VPS37A mutant livers upregulated the NRF2/NFE2L2 antioxidant pathway and exhibited expression profiles similar to those of transgenic and chemically induced mouse models of HCC. These results uncover an unappreciated role of LC3-positive phagophores in promoting the formation of insoluble protein aggregates, which may mitigate proteotoxicity and aid hepatocyte survival during cancer development.

## RESULTS

### Dissection of ESCRT-I function in autophagy by targeting the UEVL domain of VPS37A

**Figure 1.**
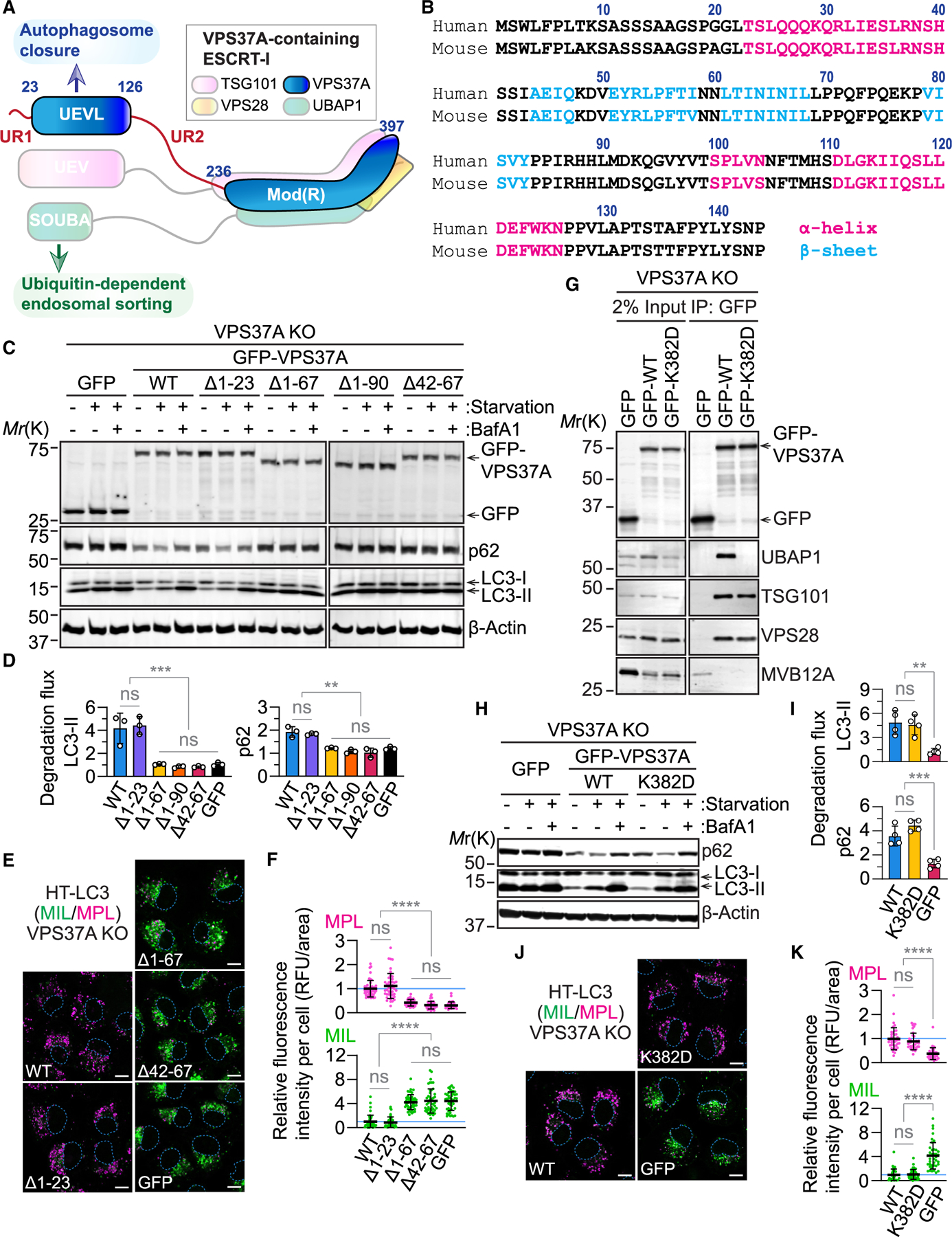
The UEVL domain of VPS37A is crucial for autophagosome closure (A) Schematic of the VPS37A-containing ESCRT-I complex. UEV, ubiquitin-E2-like variant; UEVL, UEV-like; SOUBA, solenoid of overlapping UBAs. (B) Sequences of human and mouse VPS37A N termini. (C) Immunoblot analysis of VPS37A KO U-2 OS cells that were stably transduced with the indicated constructs and starved in the presence or absence of 100 nM bafilomycin A1 (BafA1) for 3 h. (D) Bar plots of LC3-II and p62 degradation ratios ([starvation + BafA1] / starvation) in (C) (*n* = 3). (E) Confocal images of HaloTag (HT)-LC3-expressing U-2 OS cells that were starved in the presence of 100 nM BafA1 for 3 h and subjected to the HT-LC3 assay. Scale bars: 10 μm. MIL, Alexa Fluor 488-conjugated membrane-impermeable HT ligand; MPL, tetramethylrhodamine (TMR)-conjugated membrane-permeable HT ligand. (F) Dot plots of the cytoplasmic fluorescence intensities of MPL and MIL relative to the mean of GFP-WT-expressing cells in (E) (*n* = 45). (G) Immunoblot analysis of lysates (input) and immunoprecipitates (IP) from the indicated U-2 OS cells. (H) Immunoblot analysis of U-2 OS cells that were starved in the presence or absence of 100 nM BafA1 for 3 h. (I) Bar plots of LC3-II and p62 degradation ratios in (H). (J) Confocal images of U-2 OS cells that were starved in the presence of 100 nM BafA1 for 3 h and subjected to the HT-LC3 assay. Scale bars: 10 μm (K) Dot plots of the cytoplasmic fluorescence intensities of MPL and MIL relative to the mean of GFP-WT-expressing cells in (J) (*n* = 40). In (D), (F), (I), and (K), statistical significance was determined by one-way ANOVA followed by Tukey’s multiple-comparisons test. All values in the graphs are mean ± SD. **p ≤ 0.01, ***p ≤ 0.001, ****p ≤ 0.0001; ns, not significant.

Based on the AlphaFold-predicted (human: Q8NEZ2; mouse: Q8CHS8)^54^ and nuclear magnetic resonance (PDB: 8E22; BMRB: 31039)^24^ structures, the VPS37A N terminus, comprising an unstructured region (UR1) followed by an UEVL domain, is flexibly linked to the C-terminal Mod(R) domain via a long unstructured region (UR2) (Figures 1A and 1B). We have shown previously that the deletion of the first 90 residues of VPS37A impairs autophagosome closure without affecting ESCRT-I complex formation and endosomal sorting of epidermal growth factor receptor (EGFR).^7^ To gain further insight into the role of the UEVL domain in autophagosome closure, we narrowed the VPS37A N-terminal region required for autophagy by generating a series of deletion mutants. Consistent with our previous report, the Δ1–90 mutation impaired the ability of VPS37A to mediate autophagy, as determined by monitoring the lysosomal inhibitor bafilomycin A1 (BafA1)-sensitive turnover of p62 and LC3-II (autophagic flux) (Figures 1C and 1D). Similar levels of autophagic flux impairment were detected by the deletion of the first 67 amino acids (Δ1–67) or the core UEVL structure (Δ42–67) of VPS37A. In contrast, the Δ1–23 mutant restored the autophagic flux defect in VPS37A knockout (KO) cells at a level similar to VPS37A wild type (WT). These results indicate the indispensability of VPS37A UEVL, but not UR1, in autophagy. To verify that the autophagy flux defect caused by the UEVL deletion is due to the impairment of autophagosome closure, we next performed the HaloTag (HT)-LC3 autophagosome completion assay (Figures 1E and 1F). This assay utilizes a membrane-impermeable HT ligand (MIL; green) and a membrane-permeable HT ligand (MPL; magenta) conjugated with two different fluorescent dyes to distinguish open (MIL^+^MPL^−^) and closed (MPL^+^) autophagosomal membranes.^6^ Since autophagosome-sequestered HT-LC3 is subjected to lysosomal degradation, the assay was performed in the presence of BafA1. As expected, the Δ1–67 and Δ42–67 mutations, but not the Δ1–23 mutation, hindered the formation of MPL^+^ structures, resulting in an accumulation of MIL^+^ structures. This highlights the critical role of VPS37A UEVL in autophagosome closure.

Mammalian ESCRT-I is a heterotetrameric complex composed of TSG101, VPS28, one of the VPS37 homologs (VPS37A–VPS37D), and one of the UBAP1-MVB12-associated (UMA) proteins (UBAP1/1L, MVB12 A/B, and UMAD).^5^ The VPS37A-containing complex preferentially incorporates UBAP1, which contains a solenoid of overlapping ubiquitin-associated (SOUBA) domain for ubiquitin-dependent endosomal sorting (Figure 1A).^22,55^ As aberrant sorting of signaling receptors can impact autophagy,^21^ VPS37A loss or mutation may indirectly impair autophagy by disrupting the endosomal function of the complex. To test this possibility, we mutated the conserved lysine residue (K382) of VPS37A that is predicted to bind to the conserved VPF motif of UMA proteins including UBAP1.^5^ As expected, the mutation of VPS37A K382 to aspartic acid (K382D) completely abolished the incorporation of UBAP1 (Figure 1G). However, we found that the mutation did not affect both autophagosome closure and subsequent degradation, as the K382D mutant restored these autophagic events in VPS37 KO cells at levels similar to VPS37A WT (Figures 1H–1K). These results align with our previous report demonstrating the dispensability of UBAP1 in autophagosome closure^7^ and indicate that disruption of the endosomal sorting function of the VPS37A-containing ESCRT-I complex has a minimal effect on autophagosome closure.

### Establishment of an autophagosome closure-defective mouse model by targeting VPS37A UEVL

**Figure 2.**
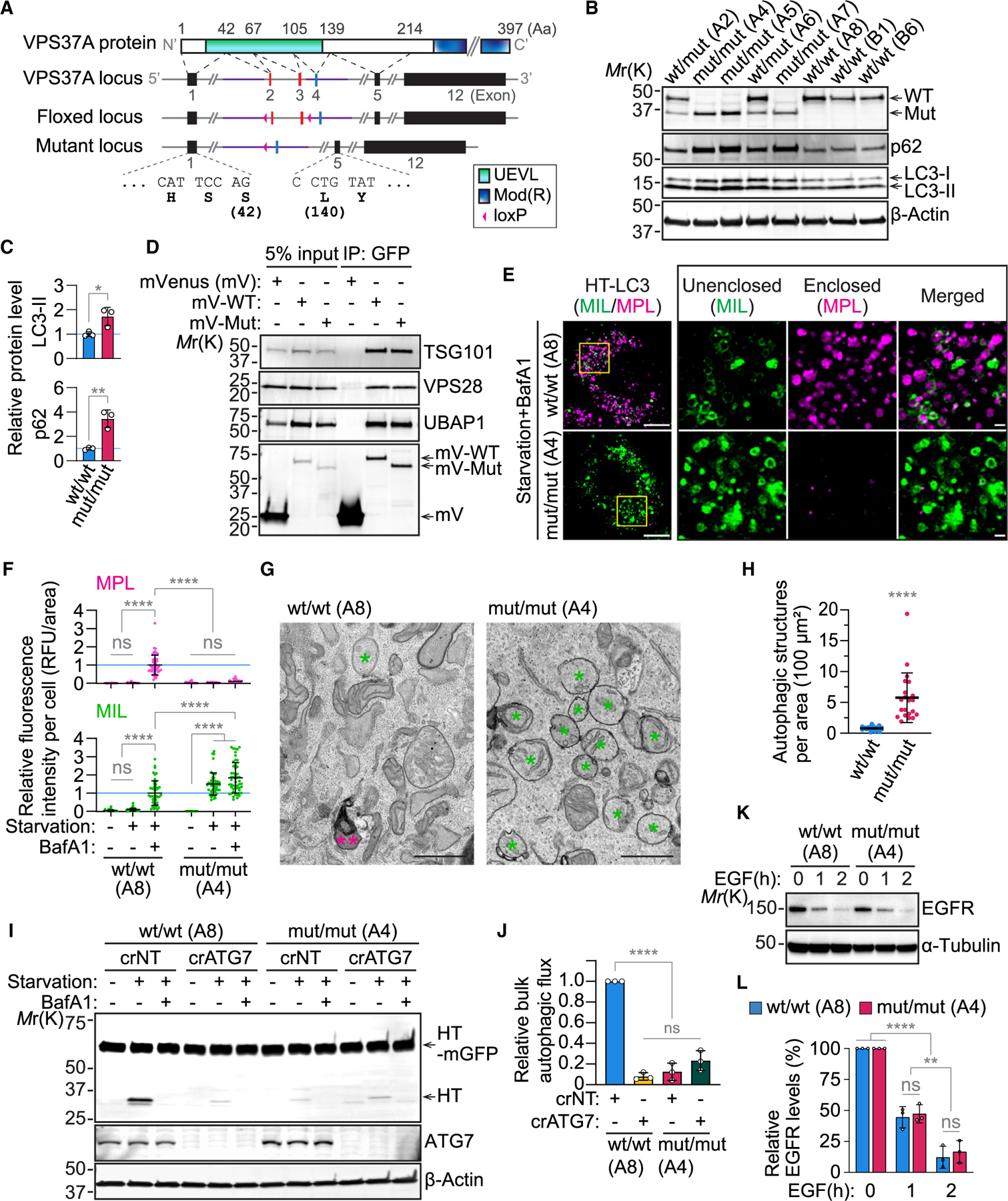
The VPS37A UEVL domain deletion mutant (Δ43–139) impairs autophagosome closure and subsequent cargo degradation (A) Schematic of the domain organization of the mouse VPS37A protein, gene locus, and targeting strategy. (B) Immunoblot analysis of primary mouse embryonic fibroblasts (MEFs) isolated from E13.5 embryos. (C) Bar plots of LC3-II and p62 levels relative to the mean of WT/WT MEFs in (B) (*n* = 3). (D) Immunoblot of lysates (input) and IPs from VPS37A KO U-2 OS cells expressing the indicated plasmids. (E) Confocal images of HT-LC3-expressing immortalized MEFs that were starved in the presence of 100 nM BafA1 for 3 h and subjected to the HT-LC3 assay. Scale bars: 10 μm and 1 μm (magnified images). (F) Dot plots of the cytoplasmic fluorescence intensities of MIL and MPL relative to the mean of WT/WT MEFs starved in the presence of BafA1 for 3 h in (E) (*n* = 50). (G) Electron micrographs of MEFs. Asterisks and a double asterisk indicate immature autophagic structures including phagophores and an autolysosome-like structure, respectively. Scale bars: 1 μm. (H) Bar plot of the number of autophagic structures per cytoplasmic area in (G) (*n* = 21). (I) Immunoblot analysis of immortalized MEFs that were stably transduced with the HT-mGFP bulk autophagic flux reporter, pulse-labeled with MPL for 20 min, and starved for 6 h in the presence or absence of 100 nM BafA1. (J) Bar plot of HT/(HT-mGFP + HT) ratio relative to WT/WT MEFs in (I) (*n* = 3). (K) Immunoblot analysis of MEFs that were serum starved for 4 h, pretreated with 10 μg/mL cycloheximide (CHX) and treated with 100 ng/mL EGF in the presence of CHX for the indicated durations. (L) Bar plot of the EGFR levels relative to respective α-tubulin in MEFs in (K) (*n* = 3). Statistical significance was determined by Student’s t test (C and H), two-way ANOVA followed by Tukey’s multiple-comparisons test (F and L), and one-way ANOVA followed by Tukey’s multiple-comparisons test (J). All values in the graphs are mean ± SD. *p ≤ 0.05, **p ≤ 0.01, ****p ≤ 0.0001.

To investigate the physiological impact of inhibiting autophagosome closure, we generated conditional VPS37A mutant C57BL/6J mice in which exons 2 and 3, encoding the core UEVL β strands, are flanked by *loxP* sites (Figure 2A). The resultant VPS37A^flox/flox^ mice were crossed with the SOX2-CRE transgenic mice (B6.Cg-*Edil3*^*Tg(Sox2-cre)1Amc*/J^)^56^ to obtain VPS37A heterozygous mutant (WT/mut) mice, which were then intercrossed to produce homozygous mutant (mut/mut) mice. Immunoblot analysis of primary mouse embryonic fibroblasts (MEFs) isolated from embryonic day 13.5 (E13.5) VPS37A^mut/mut^ embryos revealed the expression of a truncated VPS37A mutant that was accompanied by an increase in the levels of the phagophore/autophagosomal membrane marker LC3-II and the autophagic receptor p62 (Figure 2B and 2C). Minor increases in these autophagy markers were also detected in heterozygous mutants, in which both WT and mutant VPS37A proteins were detected. Sashimi plot analysis and sequencing of VPS37A transcripts verified the deletion of exons 2/3 and skipping of exon 4, resulting in the expression of a VPS37A mutant lacking the UEVL core structure (Δ43–139) (Figures S1A and S1B). Notably, the mutation did not affect the ability of VPS37A to form the heterotetrameric ESCRT-I complex with TSG101, VPS28, and UBAP1 (Figure 2D), supporting the role of its C-terminal modifier of rudimentary (Mod(r)) domain in the association with other ESCRT-I subunits.^57^ To determine the impact of the mutation on autophagosome closure, we conducted the HT-LC3 assay in immortalized MEFs. As expected, WT cells accumulated MPL^+^ structures in response to nutrient starvation in a BafA1-sensitive manner, indicating the induction of sealed and degradative autophagic vacuole formation during autophagy (Figures 2E, 2F, and S1C). Conversely, VPS37A^mut/mut^ cells hindered the formation of MPL^+^ closed autophagosomes, leading to an increase in MIL^+^MPL^−^ open phagophores. Electron microscopy confirmed the accumulation of phagophore-like immature autophagic structures in starved mutant cells (Figures 2G and 2H), demonstrating that the UEVL mutation successfully impaired autophagosome closure. To further evaluate the effect of the mutation on autophagy, we next performed the pulse-chase HT-LC3B reporter assay^58^ to monitor autophagic flux. Consistently, ATG7-dependent, BafA1-sensitive processing of HT-LC3B was found to be suppressed in VPS37A^mut/mut^ cells, although a low level of processing was still detectable (Figures S1D and S1E). This aligns with our previous finding that ESCRT-mediated autophagosome closure is crucial for efficient autolysosome formation, yet some abnormal lysosomal fusion can proceed without complete autophagosome formation.^6^ As a low level of autophagic flux is also detectable after the disruption of LC3/ATG8 conjugation,^6,58^ we next compared the inhibitory effects on autophagic flux between VPS37A mutant and ATG7 KO cells using the HT-GFP bulk degradation reporter. In line with a previous report,^58^ ATG7 loss strongly but not completely inhibited BafA1-sensitive processing of HT-GFP (Figures 2I and 2J). Notably, VPS37A^mut/mut^ cells exhibited a similar level of HT-GFP processing inhibition, which was not further enhanced by ATG7 loss, suggesting that VPS37A and ATG7 function in a linear pathway to regulate autophagic flux. The importance of VPS37A UEVL in autophagy was further verified using VPS37A KO U-2 OS human osteosarcoma cells (Figure S1F) in which the level of HT-GFP processing during autophagy was also found to be comparable to that in LC3 conjugation-defective ATG5 KO cells (Figure S1G).

A transient depletion of VPS37A has been shown to disrupt the endolysosomal system and impair ligand-stimulated degradation of EGFR.^57^ However, these effects appear to be minor or negligible for stable KO or N-terminal deletion (Δ1–90) of VPS37A.^7,59^ Consistently, the rate of EGF-stimulated endocytic EGFR degradation in VPS37A^mut/mut^ MEFs was comparable to that in WT cells (Figures 2K and 2L). Moreover, unlike the ATPase-defective VPS4A mutant (VPS4A^E228Q^), which globally perturbs ESCRT functions in various pathways, including the endolysosomal pathway, the VPS37A UEVL mutation did not affect the distribution or morphology of EEA1-positive early endosomes and LAMP1-positive late endosomes/lysosomes in MEFs (Figure S1H). Collectively, we generated a conditional mouse model expressing the autophagy-defective but endocytosis-competent VPS37A ΔUEVL mutant, which enables the investigation of the physiological and pathological implications of inhibiting autophagosome closure.

### VPS37A^mut/mut^ mice can survive but develop neuronal dysfunction

**Figure 3.**
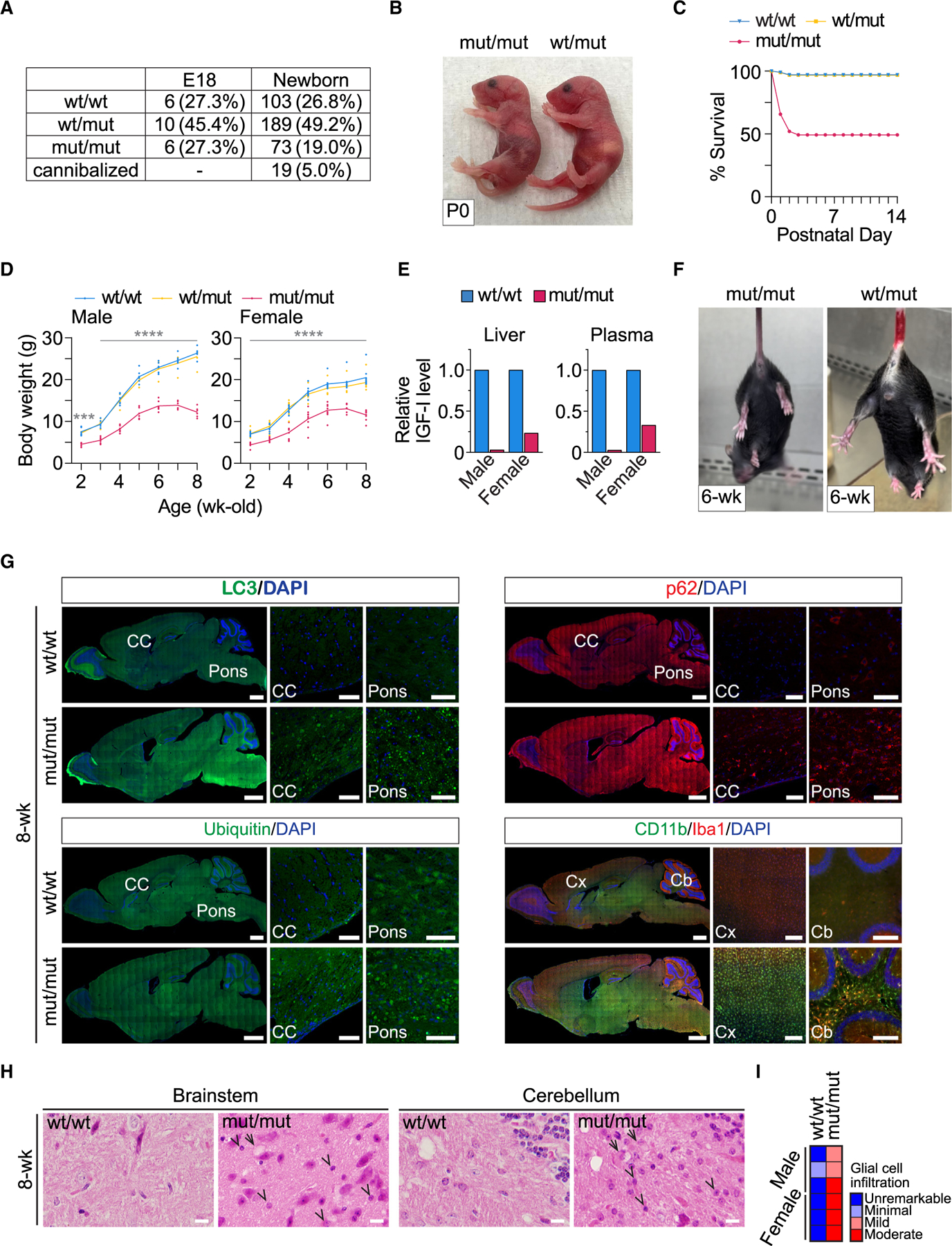
VPS37A UEVL mutant mice display neonatal lethality, growth retardation, and neurological defects (A) Numbers of mice at E18 and new born stages. (B) Images of newborn mice with the indicated genotypes. (C) Survival curve of mice. (D) Body weight measurements of male (*n* = 6) and female (*n* = 9) mice. Statistical significance was determined by two-way ANOVA followed by Tukey’s multiple-comparisons test. All values in the graphs are mean ± SD. ***p ≤ 0.001, ****p ≤ 0.0001. (E) Bar plots of liver and plasma IGF-I levels relative to WT/WT mice. (F) Images of abnormal limb-clasping and normal limb-extension reflexes in mut/mut and WT/mut mice, respectively, at the age of 6 weeks. (G) Fluorescence images of sagittal sections of 8-week-old mouse brains stained with the indicated antibodies. Nuclei were counterstained with DAPI. CC, corpus callosum; Cx, cerebral cortex; Cb, cerebrum. Scale bars: 1 mm (whole brain), 50 μm (CC and pons), and 200 μm (Cx and Cb). (H) Micrographs of H&E-stained brain stem and cerebellum sections from 8-week-old mice. Arrows and arrowheads indicate degenerating neurons and glial cell infiltration, respectively. Scale bars: 10 μm. (I) Semi-quantitative scoring of glial cell infiltration in (H).

The heterozygous mutant mice were born normally and developed into adulthood without observable abnormalities. The homozygous mutant mice were also born at the expected Mendelian frequency, when accounting for cannibalized newborns, and did not display apparent developmental defects. However, their sizes were smaller compared to WT and heterozygous littermates (Figures 3A, 3B, and S2A). Notably, approximately half of the VPS37A^mut/mut^ mice experienced neonatal lethality, with 25% dying within 1 day of birth (Figure 3C). These neonatally lethal mice often lacked milk in their stomachs (Figure 3B), suggesting a potential suckling defect. However, this alone may not explain the neonatal lethality, as an additional 25% of the VPS37A^mut/mut^ mice were found dead between post-natal days 1 and 2 (Figure 3C) despite having milk in their stomachs. These phenotypic outcomes differ from mice systemically lacking ATG genes upstream of autophagosome closure, where lethality typically occurs during *in utero* development or in the neonatal period.^10^ VPS37A^mut/mut^ mice surviving beyond day 2 showed severe growth retardation accompanied by reduced insulin growth factor I (IGF-I) plasma concentrations regardless of gender (Figures 3D, 3E, and S2B). By 2 weeks of age, all surviving VPS37A^mut/mut^ mice displayed evident neurological abnormalities, including hindlimb clasping, tremor, and gait ataxia (Figure 3F; Videos S1, S2, and S3), reaching humane endpoints within 3 months of age. These phenotypes align with those reported in LC3 conjugation-defective mice^10,13,14^ and indicate the importance of autophagic degradation in neuronal function.

To investigate the pathological alterations associated with the neurodegenerative features of the homozygous mutant mice, brain sections from 8-week-old mice were subjected to immunohistology (IH) and hematoxylin and eosin (H&E) staining. In support of the role of VPS37A UEVL in autophagosome closure for cargo degradation, IH revealed the accumulation of cytoplasmic aggregates positive for LC3, p62, and ubiquitin throughout the brain of the mutant mice (Figure 3G). We also detected increased levels of the microglia markers CD11b and ionized calcium binding adaptor molecule 1 (Iba1), indicating the development of neuroinflammation in the mutant brain. Consistent with these findings, histological analysis of H&E-stained brain sections identified degenerating neurons and glial cell infiltration in the brain stem and cerebellum (Figures 3H and 3I). To investigate the timing of neuroinflammation in the mutant mouse, we next performed IH for the astrocyte marker glial fibrillary acidic protein (GFAP) and Iba1 on E18 and 2-week-old brains. We observed elevated GFAP and Iba1 expression in the brain of VPS37A^mut/mut^ mice at 2 weeks (Figure S2C), coinciding with the onset of neurological symptoms. In contrast, there were no increases in these inflammation markers in the E18 mutant mouse brain. This discrepancy was not due to the lack of protein aggregates, as increased p62-positive foci were evident in both E18 and 2-week-old brains from the mutant mice (Figure S2D). These findings indicate that the inhibition of ESCRT-mediated autophagosome closure postnatally induces neuroinflammation and neurodegeneration.

### VPS37A UEVL loss results in tissue abnormalities accompanied by accumulation of phosphorylated p62 and ubiquitinated proteins

**Figure 4.**
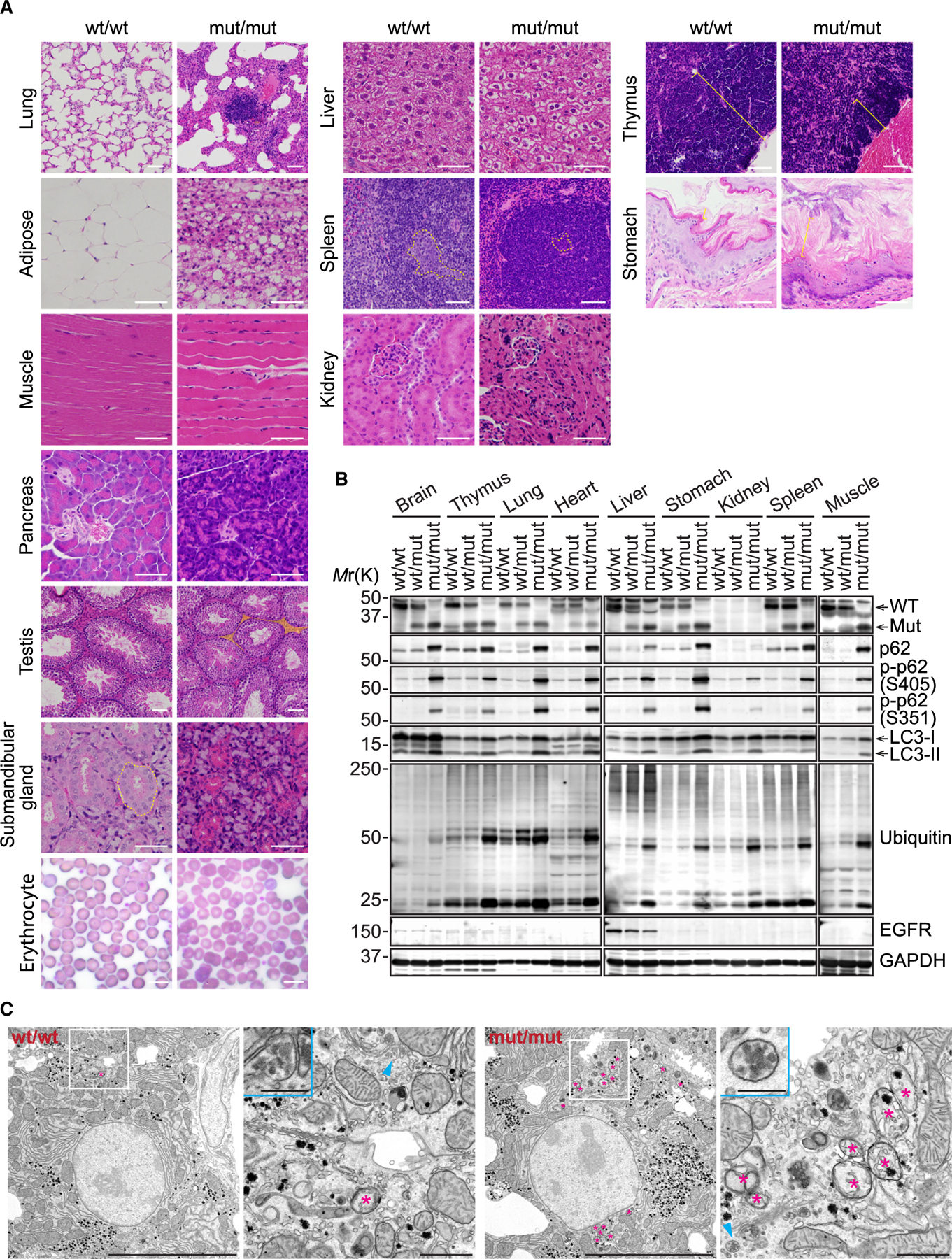
VPS37A UEVL loss causes tissue abnormalities accompanied by the accumulation of ubiquitinated proteins and phosphorylated p62 (A) Micrographs of H&E-stained tissue sections and Giemsa-stained blood smears. Dotted lines in the submandibular gland and spleen show granular convoluted ducts and germinal centers, respectively. Highlighted area in the testis show Leydig cell areas. Brackets in the thymus and stomach show the thymic cortex and mucus layer, respectively. Scale bars: 50 μm. (B) Immunoblot analysis of tissue lysates prepared from 8-week-old mice. (C) Electron micrographs of hepatocytes in 2-week-old mouse livers. Magnified images in the boxed and arrowhead-indicated areas are shown on the right and in the insets, respectively. Asterisks indicate immature autophagic structures, including phagophores. Scale bars: 10 μm, 1 μm (magnified images), and 250 nm (insets).

In accordance with their smaller body sizes, VPS37A^mut/mut^ mice exhibited reduced tissue sizes compared to their WT and heterozygous siblings at 8 weeks of age, with the exception of the lungs (Figures S3A and S3B). The tissue-to-body weight ratios were lower in the pancreas, fat, and reproductive organs (seminal vesicles, ovaries, and uterus) for VPS37A^mut/mut^ mice, while the brain and lungs had significantly higher ratios (Figure S3B). Histological analyses revealed various tissue abnormalities in VPS37A^mut/mut^ mice (Figure 4A). The lungs displayed severely thickened alveolar septa with lymphocyte infiltration and nodules of hyperplastic lymphoid tissue. The adipocytes in the fat tissues were small in size with eosinophilic cytoplasm and multiple small vacuoles, as opposed to the single large clear vacuole in WT adipocytes. The skeletal muscle contained noticeably smaller myofibers with an increased number of nuclei per myofiber. The exocrine pancreas displayed acinar cell atrophy. In the male mutants, the testes showed a large decrease in Leydig cells in the interstitial area. Although mouse submandibular salivary glands normally demonstrate sexual dimorphism histologically (with male glands having large duct epithelial cells containing eosinophilic granules), the salivary ducts of mutant male mice were as small as female salivary duct cells. Erythrocytes showed unevenly distributed sizes with central pallor. These observations are consistent with the phenotypes of LC3 conjugation-deficient mice^10–12^ and underscore the importance of autophagy in tissue development and homeostasis.

In addition to the tissue abnormalities that were characteristic of LC3 conjugation-defective mice, VPS37A^mut/mut^ mice also displayed unique phenotypes. One notable difference was the absence of hepatomegaly (Figure S3B), a prominent phenotype associated with autophagy impairment.^10–12,18,60,61^ VPS37A^mut/mut^ mice did not manifest a significant increase in the plasma level of the liver injury marker alanine aminotransferase (Figure S3C), and only mild cytoplasmic clearing was observed in their hepatocytes (Figure 4A). Moreover, the spleen showed less extramedullary hematopoiesis in the red pulp and decreased size of the white pulp with very small germinal centers and reduction in lymphoid precursors. The thymus displayed greatly reduced cortical thickness and fewer mature lymphocytes. The kidneys displayed a reduced size of the renal tubular epithelial cells, although the previously reported morphological abnormality of the glomeruli^12^ was not observed. The non-glandular stomach displayed squamous cell hyperplasia with increased thickness and prominent basal cells.

To assess whether the tissue abnormalities observed in VPS37A^mut/mut^ mice were accompanied by the impairment of autophagy, we measured the levels of ubiquitinated proteins, p62 and LC3-II, by immunoblotting. As anticipated, these markers for autophagic degradation were found to be increased in all homozygous mutant tissues examined (Figure 4B), indicating a global autophagy defect in the mice. Some heterozygous mutant tissues also showed higher levels of these indicators, consistent with the MEF data (Figure 2B). The p62 accumulation in the mutant tissues correlated with increased phosphorylation of S405 and S351, which modulate the autophagy receptor functions of p62.^62^ S403 phosphorylation of p62 (S405 in the mouse) enhances ubiquitin binding to drive phase separation for cargo concentration and segregation,^63^ while S349 (S351 in the mouse) phosphorylation enhances its binding to the signaling molecules KEAP1 and RB1CC1 for NRF2 activation and autophagy initiation, respectively.^64,65^ We detected systemic increases in both S405 and S351 phosphorylation in the mutant mice (Figure 4B). Interestingly, this observation contrasts with the data in *ATG5*^−/−^;NSE-ATG5 mice, where strong increases in phosphorylated p62 are specifically detected in the liver and skeletal muscle.^10,12^ Notably, despite these changes in autophagy markers, the levels of EGFR did not increase in the liver. Furthermore, unlike the complete depletion of VPS37A,^66^ the levels of glucagon receptor signaling pathway markers (phosphorylated, active protein kinase A [p-PKA] substrates and p-CREB) were also similar between WT and mutant livers (Figure S4). These results indicate the dispensability of the VPS37A UEVL domain for endosomal receptor sorting *in vivo*. Consistently, electron microscopy revealed the presence of endosomes with intraluminal vesicles in both WT and mutant liver sections, while accumulation of immature autophagic structures was only observed in the mutant samples (Figure 4C).

### The VPS37A UEV domain mutant liver exhibits upregulation of genes associated with antioxidant and oncogenic pathways

**Figure 5.**
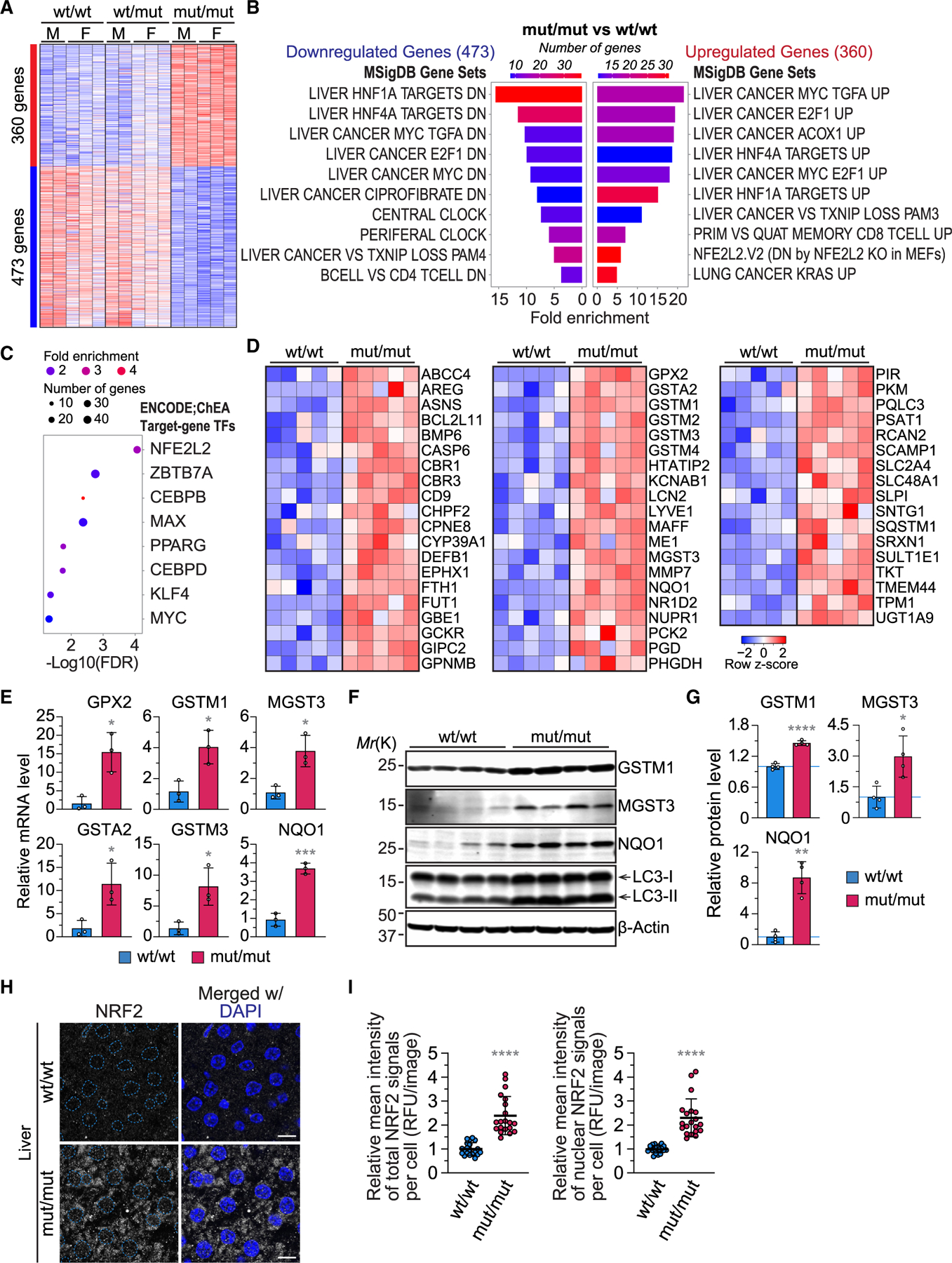
VPS37A UEVL loss stabilizes NRF2 and upregulates antioxidant genes in the liver (A) Heatmap of differentially expressed genes in the liver of 8-week-old mice with the indicated genotypes (│fold change (FC)│ ≥ 1.5; padj < 0.05). (B) Bar plots of MSigDB pathways that are enriched (right) or depleted (left) in (A) (false discovery rate [FDR] < 0.05). (C) Bar plot of the top 10 transcription factors predicted to drive expression of the 360 genes upregulated in 8-week-old mouse livers with the VPS37A UEVL mutation (FDR < 0.05). (D) Heatmap of expression of the NRF2 target genes identified in (C). (E and F) qPCR (*n* = 3) (E) and immunoblot (F) analyses of liver homogenates prepared from 8-week-old mice. (G) Bar plots of the protein levels relative to WT/WT livers in (F) (*n* = 3). (H) Confocal images of 8-week-old mouse liver sections stained for NRF2. Scale bars: 10 μm. (I) Dot plots of total and nuclear NRF2 signals per cell per field in (H) (*n* = 20). Statistical significance was determined by Welch’s t test (E and G) and Mann-Whitney nonparametric t test (I). All values in the graphs are mean ± SD. *p ≤ 0.05, **p ≤ 0.01, ***p ≤ 0.001, ****p ≤ 0.0001.

VPS37A was initially identified as a cell growth inhibitory protein frequently downregulated in HCC.^23^ The decrease in VPS37A expression has also been reported in patients with severe non-alcoholic steatohepatitis,^66^ underscoring the significance of VPS37A in maintaining liver health. To understand the impact of VPS37A-mediated autophagosome closure inhibition on tissue homeostasis, we conducted RNA sequencing on 8-week-old mouse livers with different VPS37A genotypes. Our analysis identified 797 genes that were either upregulated (360 genes) or downregulated (473 genes) in VPS37A^mut/mut^ livers compared to WT livers (│fold change (FC)│ ≥ 1.5, adjusted *p* value [padj]<0.05) (Figure 5A; Table S1). VPS37A^wt/mut^ and WT livers had similar expression patterns at this age. Gene set enrichment analysis revealed the upregulation of glucose transporter and carbohydrate metabolism-regulatory genes in VPS37A^mut/mut^ livers (Figures S5A–S5E), suggesting the activation of pathways to supply building blocks and energy to overcome autophagy deficiency. Unlike ATG7-deficient livers,^11^ only a modest enrichment of the innate immune response pathway was detected (Figures S5E and S5F), aligning with the observed liver histology. Notably, the expression patterns of both the upregulated and downregulated gene sets closely resembled those of transgenic and chemically induced mouse models of HCC^67^ in the Molecular Signatures Database (MSigDB) (Figure 5B), indicating a tumor suppressor function for VPS37A-mediated autophagosome closure.

Our analysis on MSigDB also revealed that VPS37A^mut/mut^ liver upregulates genes that are downregulated in NRF2/NFE2L2 KO MEFs (Figure 5B). Consistently, analysis of the transcription factor target dataset showed the enrichment of NRF2 target genes (Figures 5C and 5D). The upregulation of NRF2 target anti-oxidant genes in VPS37A^mut/mut^ liver was validated by qPCR and immunoblot analyses (Figures 5E–5G). These changes in gene expression were accompanied by increased NRF2 protein levels in both cytoplasm and nucleus (Figure 5H and 5I), consistent with the role of p62 in NRF2 stabilization.^15^ In addition to NRF2 target genes, we identified gene sets regulated by transcriptional factors involved in HCC development, such as MYC/MAX (Figure 5C), supporting the notion that p62 promotes HCC development via activation of the NRF2 and mTOR-MYC pathways.^68^ Collectively, we demonstrated that the inhibition of VPS37A-mediated autophagosome closure results in the upregulation of genes associated with antioxidant and oncogenic pathways in the liver. The distinct liver phenotypes observed in VPS37A^mut/mut^ and ATG5/7-deficient mice imply the involvement of other factors besides autophagy inhibition and NRF2 activation in the severe liver damage caused by ATG loss.

### LC3-conjugated phagophores facilitate the formation of insoluble p62 aggregates

**Figure 6.**
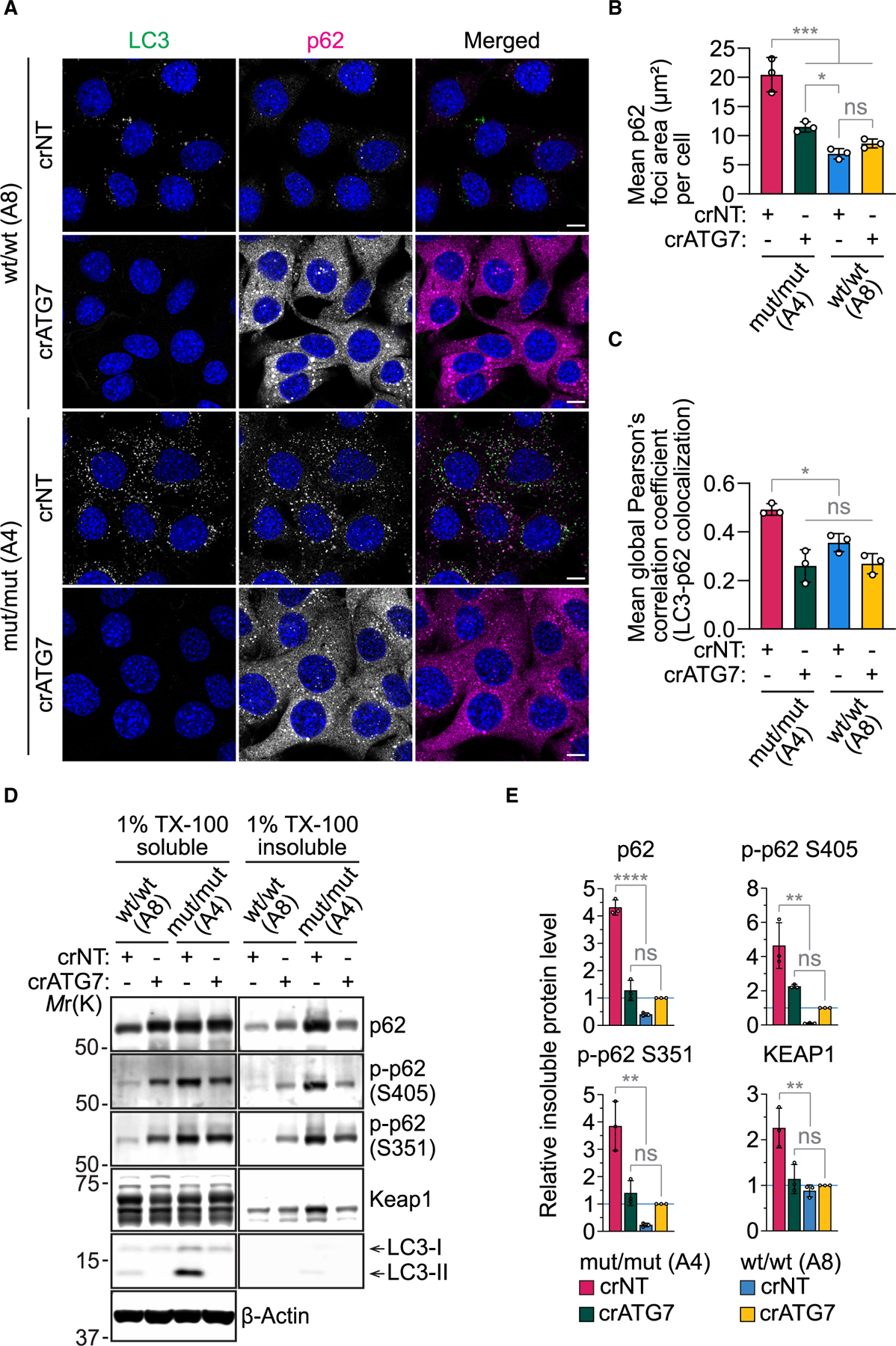
VPS37A UEVL loss promotes p62 phosphorylation and insoluble aggregates in an ATG7-dependent manner (A) Confocal images of MEFs that were starved for 3 h and stained for p62 and LC3. Scale bars: 10 μm. (B and C) Dot plots of the total p62 area (μm^2^) (B) and global Pearson’s correlation coefficient of LC3 with p62 (C) in (A) (*n* = 3). (D) Immunoblot analysis of 1% Triton X-100 (TX-100)-soluble and -insoluble fractions prepared from MEFs. (E) Bar plots of total p62, phosphorylated p62 (S405 and S351), and KEAP1 in (D) (*n* = 3). In (B), (C), and (E), statistical significance was determined by one-way ANOVA followed by Holm-Sidak’s multiple-comparisons test. All values in the bar graphs are mean ± SD. In (B) and (C), each dot represents the mean of each experiment (*n* = 20 cells per experiment). *p ≤ 0.05, **p ≤ 0.01, ***p ≤ 0.001, ****p ≤ 0.0001.

Consistent with the systemic accumulation of phosphorylated p62 in VPS37A^mut/mut^ mice (Figure 4B), we observed increased levels of S403- and S349-phosphorylated p62 in human cell lines (equivalent to S405- and S351-phosphorylated p62 in mouse cells) upon loss of VPS37A (Figures S6A–S6D). Notably, these phenotypes were not simply due to the impairment of autophagic degradation, as phosphorylated p62 levels in VPS37A-deficient cells were higher than those in ATG7-deficient cells despite similar levels of total p62 (Figures S6C and S6D). The primary cause appeared to be an autophagosome closure defect, as the phenotype is reversed by reintroducing either VPS37A WT or the K382D mutant but not by UEVL deletion mutants (Δ1–90, Δ1–90/K382D) (Figures S6E and S6F). Given that S403 phosphorylation (S405 in the mouse) of p62 reportedly promotes cytoplasmic inclusion body formation,^63^ we examined the cellular localization of p62 by confocal microscopy. In both WT MEFs and U-2 OS cells, loss of ATG7 impaired nutrient starvation-induced LC3-positive autophagosome formation and drastically increased cytoplasmic p62 signals (Figure 6A and S6G). In line with the notion that oligomerized p62 can localize to the autophagosome formation site independent of LC3 conjugation,^69^ the enhanced signals were occasionally detected in cytoplasmic puncta; however, we found that most of the p62 signals in these cells remained dispersed in the cytoplasm. In contrast, the majority of p62 signals in VPS37A^mut/mut^ and VPS37A KO cells were accumulated in cytoplasmic aggregates and associated with LC3-positive phagophores (Figures 6B and 6C). Moreover, we detected higher levels of total and phosphorylated p62 in the detergent-insoluble fraction of VPS37A^mut/mut^ cells than in ATG7 KO and WT cells (Figures 6D and 6E). The detergent-insoluble fraction of VPS37A^mut/mut^ cells was also enriched with the E3 ubiquitin ligase KEAP1, supporting the role of p62 S351 phosphorylation in KEAP1 sequestration for NRF2 stabilization.^65^ Notably, the increased p62 phosphorylation and inclusion formation in VPS37A mutant and KO cells were not further enhanced but reversed by ATG7 deletion (Figures 6A, 6B, 6D, 6E, S6C, S6D, and S6G). These results indicate the roles of LC3-conjugated phagophores in promoting p62 phosphorylation and inclusion formation upon autophagy inhibition.

### Phagophore-associated TBK1 mediates p62 phosphorylation and augments insoluble inclusion formation

**Figure 7.**
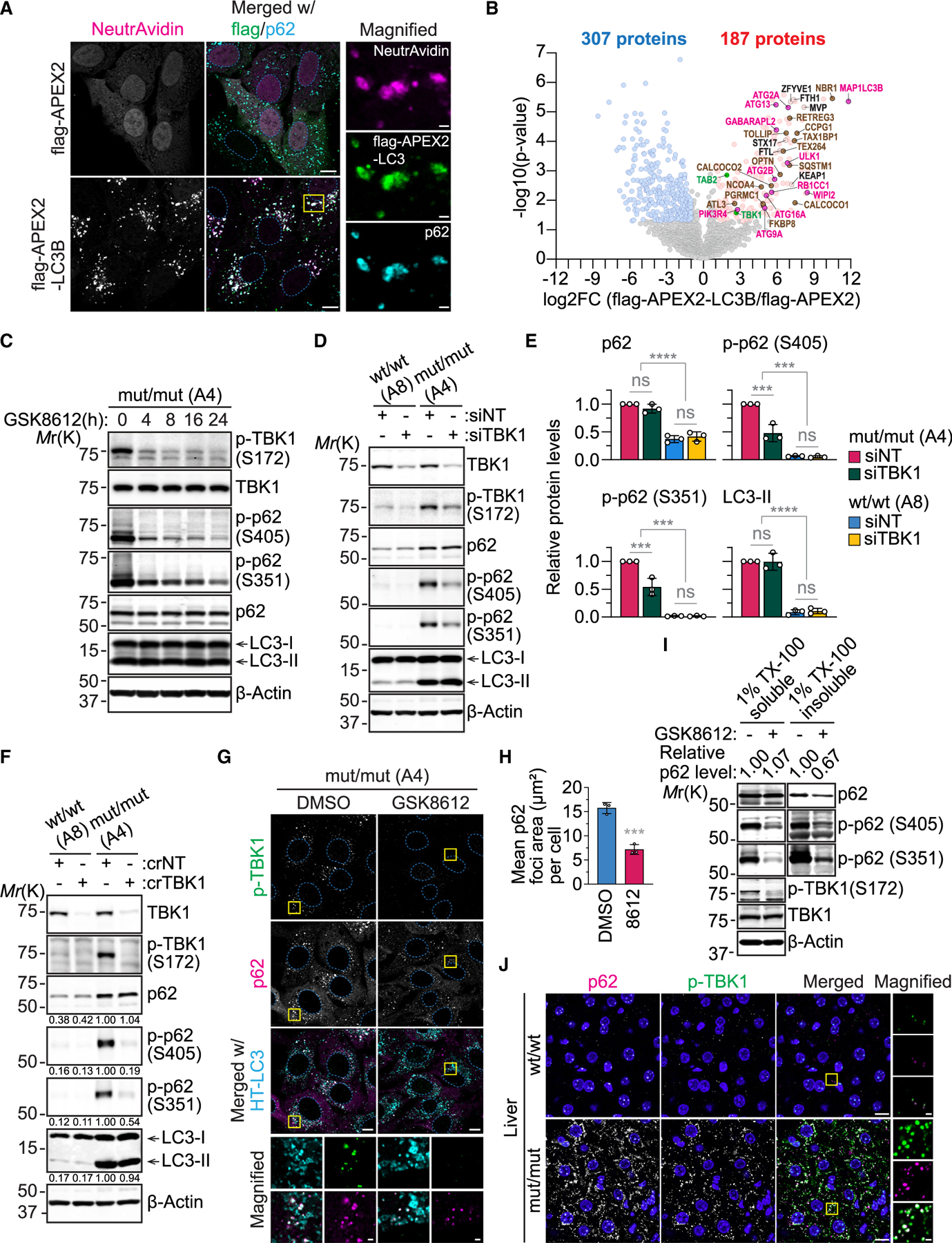
TBK1 is responsible for p62 phosphorylation and inclusion formation enhanced by VPS37A UEVL loss (A) Confocal images of the VPS37A KO U-2 OS cells transduced with FLAG-APEX2 control or FLAG-APEX2-LC3B, preincubated with biotin phenol and H_2_O_2_, followed by DyLight 650-conjugated NeutrAvidin incubation, and stained for FLAG and p62. Scale bars: 10 μm and 1 μm (magnified images). (B) Volcano plot of proteins enriched by FLAG-APEX2-LC3B WT vs. FLAG-APEX2 control. Significantly enriched proteins are colored (*p* < 0.05). (C) Immunoblot analysis of MEFs treated with 10 μM GSK8612 for the indicated periods of time. (D) Immunoblot analysis of MEFs that were transiently transfected with TBK1 siRNA (siTBK1) or control non-targeting siRNA (siNT) for 72 h. (E) Bar plots of the indicated protein levels relative to the siTBK1-transfected mut/mut MEFs in (D) (*n* = 3). (F) Immunoblot analysis of MEFs that were stably transduced in combination with Cas9 and either non-targeting or TBK1 single guide RNAs. (G) Confocal images of HT-LC3-expressing MEFs treated with 10 μM GSK8612 for 48 h, pre-stained with MPL-TMR for 15 min, and stained for p62 and p-TBK1. Scale bars: 10 μm and 1 μm (magnified images). (H) Bar plot of total p62 area (μm^2^) in (G) (*n* = 3). (I) Immunoblot analysis of 1% TX-100-soluble and -insoluble fractions prepared from mut/mut MEFs incubated in complete medium in the presence or absence of 10 μM GSK8612 for 48 h. p62 levels in TX-100-soluble and TX-100-insoluble fractions relative to untreated MEFs are shown. (J) Confocal images of 8-week-old mouse liver sections stained for p62 and *p*-TBK1. Scale bars: 10 μm and 1 μm (magnified images). Statistical significance was determined by one-way ANOVA followed by Holm-Sidak’s multiple-comparisons test (E) and Mann-Whitney nonparametric t test (H). All values in the bar graphs are mean ± SD. In (H), each dot represents the mean of each experiment (*n* = 20 cells per experiment). ***p ≤ 0.001, ****p ≤ 0.0001.

To understand the mechanism underlying the augmentation of phagophore-dependent p62 phosphorylation observed by autophagosome closure inhibition, we utilized the enhanced ascorbate peroxidase 2 (APEX2)-mediated proximity biotinylation approach^70^ to label proteins on LC3-positive membranes that accumulated in VPS37A KO U-2 OS cells. The successful enrichment of biotinylated proteins on p62-positive phagophores by FLAG-APEX2-LC3B, as opposed to control FLAG-APEX, was confirmed through NeutrAvidin staining (Figure 7A). To identify biotinylated proteins in close proximity to LC3-positive phagophores, cell lysates were subjected to streptavidin pull-down followed by mass spectrometry. This revealed 494 proteins either enriched (187 proteins) or depleted (307 proteins) by FLAG-APEX2-fused LC3B over FLAG-APEX (log2 fold change [log2FC] > 1, *p* < 0.05) (Figure 7B; Table S2). Analysis using the COMPARTSMENTs protein subcellular localization database demonstrated strong enrichment for phagophore and autophagosomal membrane-associated proteins, including 11 core ATG proteins (ULK1, ATG13, RB1CC1, PIK3R4, ATG2A/B, ATG9A, WIPI2, ATG16L, MAP1LC3B, and GABARAPL2); the omegasome marker ZFYVE1; and 14 autophagy receptors, including p62/SQSTM1; as well as known autophagic substrates like KEAP1, FTH1, FTL, and MVP by FLAP-APEX2-LC3 (Figures 7B, S7A, and S7B). We also detected increases in proteins involved in membrane tethering and fusion, such as STX17, as well as upstream ESCRT factors, including TOM1 and TOM1L1. The 307 proteins enriched by FLAP-APEX over FLAG-APEX2-LC3 included many nucleoproteins, reflecting the cytoplasmic and nuclear distribution of FLAG-APEX (Figure 7A).

Among the 173 APEX2-LC3-enriched proteins, several serine/threonine kinases (MST1, NEK9, PLK1, TBK1, and ULK1) and kinase adaptors (AZI2 for TBK1 and TAB2 for TAK1) were identified. Notably, TAK1, TBK1, and ULK1 have been implicated previously in S403/405 and/or S351/349 phosphorylation of p62.^41,71–74^ To investigate their involvement in upregulation of p62 phosphorylation during autophagosome closure inhibition, we treated VPS37A^mut/mut^ cells with the TBK1 inhibitor GSK8612,^75^ the ULK1 inhibitor SBI-0206965,^76^ and the TAK1 inhibitor 5Z-7-oxozeaenol.^77^ GSK8612 treatment resulted in a time-dependent reduction of S172-phosphorylated (active) TBK1 and S405-phosphorylated p62 without affecting total p62 and LC3-II (Figure 7C). The inhibitory effect of GSK8612 on p62 phosphorylation appeared to be specific to TBK1 inhibition, as similar results were obtained by small interfering RNA (siRNA)- and CRISPR-Cas9-mediated depletion of TBK1 (Figures 7D–7F). A similar trend was observed for p62 phosphorylation at S351, albeit less pronounced than at S405 (Figures 7C–7F). This is in line with previous studies suggesting that S405/403 phosphorylation can be directly mediated by TBK1^71,74^ and is important for the subsequent phosphorylation at S351/349.^78^ The upregulation of TBK1-mediated p62 phosphorylation by autophagosome closure inhibition was further validated in VPS37A-deficient U-2 OS, HuH-7, and Hep G2 cells (Figure S8A). In contrast, SBI-0206965 and 5Z-7-oxozeaenol showed little inhibitory effects on p62 phosphorylation, although they reduced other downstream targets, including ATG13 phosphorylation and tumor necrosis factor alpha-induced IkB degradation, respectively (Figures S8B and S8C). We next examined whether TBK1 activation is responsible for the increased formation of p62 inclusions in autophagosome closure-defective cells. We found that p62 inclusions accumulated in VPS37A^mut/mut^ and VPS37A KO cells were highly associated not only with LC3 but also with phosphorylated TBK1 and that GSK8612 treatment abrogated inclusion formation (Figures 7G and 7H). Consistently, cell fractionation analysis revealed that TBK1 inhibition decreased the levels of not only phosphorylated but also total p62 proteins in the detergent-insoluble fraction (Figure 7I). A drastic accumulation of active TBK1-positive p62 inclusions was also detected in the liver of VPS37A^mut/mut^ mice (Figure 7J). Notably, the phagophore accumulation of active TBK1 was found to be dependent on LC3 conjugation, as ATG7 depletion dispersed p-TBK1 signals in VPS37A^mut/mut^ cells (Figures S8D and S8E). Collectively, these results indicate that LC3-conjugated phagophores serve as a platform for TBK1 to phosphorylate p62, mediating inclusion formation, which is amplified upon phagophore closure inhibition.

## DISCUSSION

In this study, we developed a mouse model of defective autophagosome closure by targeting the core UEVL structure of VPS37A and investigated its physiological impact *in vivo*. VPS37A^mut/mut^ mice expressing the UEVL-truncated VPS37A (Δ43–139) mutant exhibited impaired autophagosome closure and cargo degradation, resulting in phenotypes characteristic of autophagy-defective mice. These phenotypes included the accumulation of p62 and ubiquitinated proteins, neuronal dysfunction, growth retardation, and tissue abnormalities.^10^ Importantly, despite these autophagy-related effects, the VPS37A (Δ43–139) mutant retained the ability to form a heterotetrameric ESCRT-I complex with TSG101, VPS28, and UBAP1 and to regulate endosomal receptor sorting. Consequently, VPS37A^mut/mut^ mice did not exhibit the embryonic developmental abnormalities or lethality observed in endosomal sorting-defective UBAP1 mutant and TSG101 KO mice.^19,20^ These findings extend our previous study^7^ and underscore the critical role of the VPS37A UEVL domain in mammalian autophagosome closure.

Although VPS37A^mut/mut^ mice developed autophagy-defective phenotypes, they also displayed distinct features compared to mice with defective LC3 conjugation. One major difference is the neonatal mortality rate. While nearly all LC3-conjugation-defective mice die on post-natal day 0, primarily due to suckling failure,^10,60,79^ only half of the VPS37A^mut/mut^ mice died during the neonatal period, with the rest surviving for more than 2 weeks. Moreover, even among VPS37A^mut/mut^ mice that died as neonates, about half of them exhibited signs of milk intake. These phenotypes mirror the rescue of the neonatal lethality of ATG5-null mice by neuron-specific transgenic expression of ATG5,^12^ suggesting that the presence of LC3-conjugated phagophores may delay neuronal dysfunction caused by autophagy deficiency. Another notable difference was the absence of severe hepatic injury and hepatomegaly in VPS37A^mut/mut^ mice. We found that this was not due to the lack of p62 accumulation or NRF2 stabilization, both of which have been implicated in the liver pathology caused by ATG7 loss.^15,80^ Notably, nearly all p62 inclusions accumulated in VPS37A mutant or KO cells were associated with LC3-conjugated phagophores. Moreover, while small soluble aggregates are known to be highly reactive and more toxic to cells compared to large aggregates,^81,82^ we found that disruption of LC3 conjugation attenuated p62 inclusion formation in cells defective in ESCRT-mediated autophagosome closure. Thus, LC3-conjugated phagophores may have a protective role in reducing proteotoxicity resulting from autophagy deficiency, although further investigation is required to elucidate the cytoprotective roles of autophagosomal membranes. Interestingly, mice deficient in EPG5, which functions downstream of autophagosome closure to regulate autophagosome-endosome/lysosome fusion, have also been shown to survive postnatally.^83^ Notably, while these mice exhibit growth retardation and develop neuronal abnormalities, their phenotypes appear to be less severe than those observed in VPS37A mutant mice, suggesting that accumulation of unclosed and closed autophagosomes may have different degrees of protection from proteotoxicity caused by autophagy deficiency.

Our study identified the innate immune kinase TBK1 as a major kinase responsible for the increases in p62 phosphorylation and detergent-resistant inclusion formation upon autophagosome closure inhibition. TBK1 has been shown to translocate to autophagosome formation sites, where it regulates the formation and maturation of autophagosomes by phosphorylating ubiquitin-binding autophagy cargo receptors such as p62 and other autophagy regulators.^71,74,84,85^ Mechanistically, TBK1 can be recruited to LC3-conjugated phagophores either directly, through interactions with the ATG8 family protein GABARAP^86^ and the autophagy receptors OPTN and TAX1BP1,^86–88^ or indirectly via the autophagy receptor CALCOCO2/NDP52 and the TBK1 adaptor protein AZI2.^89^ Through AZI2, TBK1 can also interact with the ULK1 complex component RB1CC1/FIP200.^88^ Consistently, we detected co-enrichment of these TBK1 recruiting factors with p62 in close proximity to LC3-conjugated phagophores (Figure 7B; Table S2). Interestingly, TBK1 also phosphorylates stimulator of interferon genes (STING) to induce IRF3-mediated immune response, while autophagy degrades active TBK1 and STING to limit its downstream signaling.^90–93^ Moreover, the VPS37A-containing ESCRT-I complex has been reported recently to regulate TBK1 signaling by inducing STING degradation via endosomal microautophagy.^94,95^ However, despite the accumulation of active TBK1, severe inflammation was not observed in the VPS37A^mut/mut^ livers. Notably, our data show that phagophore closure inhibition can enhance TBK1 activation independent of the STING pathway, as VPS37A loss accumulated phosphorylated TBK1 in U-2 OS cells, which are deficient in STING.^96^ It would be of interest to determine the precise mechanisms behind TBK1 activation upon autophagosome closure inhibition and to identify its novel substrates and signaling pathways on the phagophore.

While acute liver injury was not evident, we found that VPS37A^mut/mut^ livers upregulated glucose transporter and carbohydrate metabolism-regulatory genes and displayed expression profiles characteristic of those in transgenic and chemically induced mouse models of HCC.^67^ These observations are in line with the fact that VPS37A downregulation is commonly detected in major solid cancer types, including HCC, as a consequence of chromosome 8p deletion^29,30^ and that it enhances tumor progression.^29,31,38,97^ In contrast to ATG5/7 loss, which causes severe hepatic damages but does not result in malignant tumor formation,^16^ overexpression of p62 has been shown to induce hepatocellular carcinogenesis by promoting the survival of HCC-initiating cells in mice.^68^ Moreover, increased p62 activates a positive feedback loop to further upregulate its expression through the NRF2 and nuclear factor κB axes.^98^ Thus, while cells with VPS37A/8p loss may be capable of managing acute proteotoxicity by promoting LC3 conjugation-dependent inclusion formation and sequestration, activation of the p62 feedforward loop and its associated oncogenic signaling pathways may facilitate them to undergo malignant transformation and grow further in the absence of autophagic degradation.

In summary, we established the VPS37A UEVL mutant mouse as an animal model of defective autophagosome closure and revealed an unappreciated role of LC3-conjugated phagophores in regulating the formation of insoluble protein aggregates through TBK1-mediated p62 phosphorylation. The short lifetime of phagophores hampers our understanding of the phagophore-associated signaling pathways. Given that targeting VPS37A UEVL can stabilize and enrich phagophores without apparent impact on other ESCRT-dependent membrane remodeling processes, this mouse model provides a valuable resource to facilitate the study of phagophore-dependent signaling pathways and the physiological and pathological roles of autophagosomal membranes beyond degradation.

### Limitations of the study

In this study, we demonstrated that the VPS37A UEVL mutation impairs autophagosome closure without disrupting ESCRT-I complex formation and endocytic receptor sorting, the most well-characterized non-autophagic function of VPS37A.^57^ However, we cannot exclude the possibility that the phenotypes observed in the VPS37A mutant mice may be partly attributed to disruptions of uncharacterized non-autophagic functions of VPS37A UEVL. Additionally, since ESCRT inhibition does not completely block autophagic flux,^6–8^ a residual level of autophagic degradation in the mutant cells may also contribute to the postnatal survival of the mutant mice. Finally, we acknowledge that further studies are necessary to experimentally validate the cytoprotective role of LC3-conjugated phagophores.

## RESOURCE AVAILABILITY

### Lead contact

Requests for further information, resources, and reagents should be directed to and will be fulfilled by the lead contact, Yoshinori Takahashi (ytakahashi@pennstatehealth.psu.edu).

### Materials availability

All unique plasmids and mouse lines generated in this study are available from the lead contact with a completed materials transfer agreement.

### Data and code availability

- The mass spectrometry proteomics data have been deposited to the ProteomeXchange Consortium via the PRIDE^99^ partner repository with the dataset identifier PXD057301 and https://doi.org/10.6019/PXD057301.
- This paper does not report original code.
- All other relevant information is available upon request.

## STAR★METHODS

### EXPERIMENTAL MODEL AND STUDY PARTICIPANT DETAILS

#### Mice

Heterozygous VPS37A targeted (wt/flox) C57BL/6J mice in which exons 2 and 3, encoding the core UEVL β-strands, are flanked by *LoxP* sites, were generated by Cyagen Biosciences using CRISPR/Cas9-assisted homologous recombination. Briefly, mouse genomic fragments containing 5′ and 3′ homology arms were amplified from BAC clone (RP24–74J7) and assembled into a targeting vector containing *loxP* recombination site. gRNAs (ACCCGCCACTAGCAGCTTTACGG and ATTGTCCAAAGGTAAATAATTGG) targeting mVPS37A gene, the targeting vector and Cas9 mRNA were co-injected into fertilized mouse eggs to generate targeted knock-in offspring. The VPS37A^wt/flox^ mice were intercrossed to generate homozygous VPS37A targeted (flox/flox) mice. The resultant VPS37A^flox/flox^ mice were bred with SOX2-CRE [B6.Cg-Edil3Tg(Sox2-cre)1Amc/J] transgenic mice (008454; Jackson Laboratory) to obtain VPS37A heterozygous mutant (wt/mut) mice, which were then intercrossed to produce homozygous mutant (mut/mut) mice. Genotyping of mice was performed by PCR using the primers listed in Table S3. All animal studies were performed in accordance with guidelines established by the Institutional Animal Care and Use Committee (IACUC # PRAMS201145989) at the Penn State College of Medicine (Hershey, Pennsylvania, USA).

#### Cell culture, viral transduction, and autophagy induction

Mouse embryonic fibroblasts (MEFs) were isolated from E13.5 embryos and maintained in Dulbecco’s Modification of Eagle’s Medium (DMEM) (10–013-CV; Corning) supplemented with 10% fetal bovine serum (FBS) and 1x Antibiotic Antimycotic Solution (AA) (SV30079.01; Cytiva). 293T/17 (CRL-11268), Phoenix-AMPHO (CRL-3213), HuH-7 cells (CVCL_0336, kindly gifted by Dr. Jianming Hu) and Hep G2 cells (CVCL_0027, HB-8065; ATCC) were maintained in DMEM containing 10% FBS and 1x AA. U-2 OS cells were maintained in McCoy’s 5A medium (10–050-CV; Corning) supplemented with 10% FBS and 1x AA. HuH-7 cells and Phoenix-AMPHO were gifted from Jianming Hu (Penn State College of Medicine) and Garry P. Nolan (Stanford University School of Medicine). All other human cell lines were obtained from American Type Culture Collection. Lentivirus-mediated and retrovirus-mediated gene transduction were performed as previously described.^7^ To immortalize MEFs, primary MEFs were transduced with retrovirus containing pBABE-neo largeT cDNA and selected with 400 μg/mL G418 for 7 days. VPS37A KO, ATG7 KO, ATG7 VPS37A DKO U-2 OS cells were generated as previously described.^6,7,107^ All other knockout cells were generated by lentiviral transduction with gRNAs listed in Table S3 followed by puromycin selection (4 μg/mL for MEFs; 3 μg/mL for HuH-7 and Hep G2 cells) for 10 days. For ATG7-deficient MEFs, six knockout clones were picked and pooled. For transient gene silencing, siRNAs were transfected into cells using the Lipofectamine RNAiMAX transfection reagent according to the manufacturer’s protocol. To induce autophagy, cells were rinsed three times with Dulbecco’s phosphate buffered saline (PBS) and incubated with amino acid-free DMEM (048–33575; FUJIFILM Wako Pure Chemical Corporation).

### METHOD DETAILS

#### Reagents

The following primary antibodies were used for immunoblotting (IB), immunofluorescence (IF) and immunohistology (IH): mouse antibodies against α-tubulin (IB, T5168, 1:4000; Sigma-Aldrich), β-actin (IB, A5441, 1:10,000; Sigma-Aldrich), FLAG (IH, F1804, 1:500; Sigma-Aldrich), IκBα (IB, 1:1000; Cell Signaling Technology), LC3B (IH, M152–3, 1:200; MBL international), TSG101 (IB, GTX70255, 1:1,000; Genetex), Ubiquitin (NB300–130, 1:2000 for IB, 1:300 for IH; Novus), VPS28 (IB, sc-166537, 1:100; Santa Cruz Biotechnology); rabbit antibodies against Atg5 (IB, 12994, 1:1000; Cell Signaling Technology), Atg7 (IB, 8558, 1:1000; Cell Signaling Technology), Atg13 (IB, 13273, 1:1000; Cell Signaling Technology), EEA1 (IH, MA514794,1:200; Thermo Fisher Scientific), EGFR (IB, 4267, 1:1000; Cell Signaling Technology), GFP (IB, 2956, 1:1000; Cell Signaling Technology), Gstm1 (IB, 12412–1-AP, 1:1,000; Proteintech), HaloTag (IB, G928A, 1:1000; Promega), Iba1 (IH, 019–19741, 1:200; FUJIFILM Wako Pure Chemical Corporation), Keap1 (IB, 10503–2-AP, 1:2000; Proteintech), MAP1LC3B (IB, NB100–2220, 1:4000; Novus), MGST3 (IB, ab192254, 1:1000; Abcam), MVB12A (IB, 50–173-5082, 1:1,000; Fisher scientific), NQO1 (IB, 11451–1-AP, 1:2,000; Proteintech), NRF2 (IH, 12721, 1:200; Cell Signaling Technology), phospho-Atg13 Ser355 (IB, 46329, 1:1000; Cell Signaling Technology), phospho-p62 Ser349 (IB, 95697, 1:1000; Cell Signaling Technology), phospho-p62 Ser403 (IB, 39786, 1:1000; Cell Signaling Technology), phospho-TBK1 (5483, 1:1000 for IB, 1:200 for IH; Cell Signaling Technology), TBK1 (IB, 38066, 1:1000: Cell Signaling Technology), UBAP1 (IB, 12385–1-AP, 1:1,000; Proteintech), VPS37A (IB, HPA024705, 1:4000; Sigma-Aldrich; IB, 11870–1-AP, 1:2000; Proteintech); chicken antibody against GFAP (IH, CPCA-GFAP, 1:1000; EnCor Biotechnology); guinea pig antibodies against p62 (03-GP62-C, 1:4,000 for IB, 1:300 for IH; American Research Products); rat antibodies against CD11b (IH, MCA711, 1:200; Bio-Rad), LAMP1 (IH, sc-19992, 1:100; Santa Cruz Biotechnology); DyLight 650-conjugated NeutrAvidin (IH, 84607, 1:1000; Thermo Fisher Scientific). The following secondary antibodies were used for IH: Alexa Fluor 488-conjugated goat antibodies against mouse IgG (1:1000, A28175; Life Technologies), rabbit IgG (1:1000, A11008; Life Technologies); Alexa Fluor 568-conjugated goat antibodies against rabbit IgG (1:1000, A11036; Life Technologies); Alexa Fluor 647-conjugated goat antibodies against rat IgG (1:1000, A21247; Life Technologies), guinea pig IgG (1:1000, A21450; Life Technologies). The following secondary antibodies were used for IB: HRP-conjugated goat antibodies against mouse IgG (1:3000, 7076; Cell Signaling Technology), rabbit IgG (1:3000, 7074; Cell Signaling Technology); IRDye 680RD-conjugated donkey antibodies against mouse IgG (1:10000, 926–68072; LI-COR), guinea pig IgG (1:10000, 926–68077; LI-COR); IRDye 800CW-conjugated donkey antibodies against rabbit IgG (IB, 1:10000, 926–32213; LI-COR). All other reagents were obtained from the following sources: 5Z-7-oxozeaenol (HY-12686; MedChemExpress), AlexaFluor 488-conjugated membrane-impermeable ligand (MIL) (G1001; Promega), BafA1 (AAJ61835MCR; Thermo Fisher Scientific), cycloheximide (CHX) (C7698; Sigma-Aldrich), digitonin (D141; Sigma-Aldrich), EGF from murine submaxillary gland (E4127; Sigma-Aldrich), GSK8612 (HY-111941; MedChemExpress), mouse recombinant TNFα (CYT-252; Prospec), normal goat serum (G9023; Sigma-Aldrich), paraformaldehyde (PFA) (15710, Electron Microscopy Sciences), phosphatase inhibitor cocktail 2/3 (P5726/P0044; Sigma-Aldrich), protease inhibitor cocktail (P8340; Sigma-Aldrich), protease inhibitor cOmplete mini (Sigma-Aldrich; 11836153001), SBI-0206965 (M60268; Xcess biosciences), tetramethylrhodamine-conjugated membrane-permeable ligand (MPL) (G8251; Promega), XF Plasma Membrane Permeabilizer (XF-PMP) (102504–100; Agilent). ON-TARGETplus SMART Pool Non-targeting (D-001810–10) and mouse TBK1 (L-063162–00) siRNAs were obtained from GE Healthcare Dharmacon. The following plasmids were obtained through Addgene: lentiCRISPRv2 puro (gifted from Dr. Brett Stringer, #98290),^104^ mVenus-C1 (gifted from Dr. Steven Vogel, #27794),^101^ pEGFP-flag-APEX2-α-tubulin (gifted from Dr. Alice Ting, #66171),^70^ pEGFP-VPS4-E228Q (gifted from Dr. Wesley Sundquist, #80351),^103^ pMRX-IB-HaloTag7-mGFP (gifted from Dr. Noboru Mizushima, #184903),^58^ pBABE-neo largeT cDNA (gifted from Dr. Robert A Weinberg, #1780),^102^ FUGW-PK-hLC3 (gifted from Dr. Isei Tanida, #61460).^100^ The oligonucleotides used for plasmid construction are listed in Table S3. pCDH1-CMV-MCS-SV40-Hygro and pCDH1-CMV-HT-LC3-SV40-Hygro were generated as previously described.^7^ pCDH1-Ubc-HT-LC3B was generated by subcloning the PCR-amplified HT-LC3 cassette into NheI–EcoR1 site of FUGW-PK-hLC3. To make pCDH-mVenus-Hygro, mVenus from mVenus-C1 was amplified by PCR and subcloned into Xbal-BamHI site of pCDH1-MCS-SV40-Hygro by Gibson assembly. PCR-amplified hVPS37A was subcloned into XhoI-BamHI site of pCDH1-CMV-HT-LC3-SV40-Hygro, and the HT cassette in the NheI-XhoI site of resultant pCDH-HT-hVPS37A was replaced with mVenus cassette to generate pCDH-mVenus-hVPS37A. For mVPS37A expression constructs, total RNA from mouse liver was extracted using RNeasy plus mini kit (74134; Qiagen), and cDNA were generated by QuantiTect Reverse Transcription Kit (205311; Qiagen). mVPS37A FL and mutant lacking the UEVL core structure (Δ43–139) were amplified by PCR and cloned into NheI-XhoI sites of pCDH-mVenus-hVPS37A to generate pCDH-mVenus-mVPS37A FL (WT) and pCDH-mVenus-mVPS37A Δ43–139 (Mut), respectively. pCDH-flag-APEX2-LC3 was generated by subcloning of flag-APEX2 amplified from pEGFP-flag-APEX2-α-tubulin and LC3B amplified from pCDH1-CMV-HT-LC3-SV40-Hygro into the XbaI-BamhI site of pCDH-CMV-MCS-EF1α-Puro (#CD510A-1; System Biosciences). To make an inducible dominant-negative (DN) VPS4 (VPS4A^E228Q^) expression plasmid, pCDH-TRE3G-GFP-DNVPS4 was generated by subcloning the tetracycline operator and GFP-DNVPS4A from pEGFP-VPS4-E228Q into the ClaI-BamHI site of pCDH-CMV-MCS-EF1α-Puro. Tandem P2A-T2A and tetracycline-controlled transactivator were subcloned into SalI site of pCDH-TRE3G-GFP-DNVPS4. Finally, CMV enhancer amplified from pCDH-Cuo-MCS-EF1α-CymR-T2A-Puro (#QM800A-1; System Biosciences) was subcloned into the ClaI site of the resultant plasmid to generate pCDH-TRE3G-GFPDNVPS4-tet-on-3G. Each gRNA listed in Table S3 were subcloned into the BsmB1 site of lentiCRISPRv2.

#### Immunoblotting and coimmunoprecipitation

Total cell and tissue lysates were prepared in radio-immunoprecipitation assay buffer (RIPA buffer, 150 mM NaCl, 10 mM Tris-HCl, pH 7.4, 0.1% sodium dodecyl sulfate (SDS), 1% Nonidet P-40, 1% Deoxycholate, 5 mM EDTA, pH 8.0) containing phosphatase inhibitor cocktail 2/3 and protease Inhibitor cocktail, and subjected to SDS-PAGE followed by immunoblotting with the indicated antibodies. For tissue lysates, tissues were homogenized in RIPA buffers using a Polytron 1300D homogenizer at 13,000 rpm for muscle and 5,000 rpm for the other tissues with 5 up-and-down strokes followed by incubation on ice for 30 min. The signal intensities were quantified using Image Studio software (version 5.2, LI-COR Biotechnology). For coimmunoprecipitation, cell lysates were prepared in 0.5% NP-40 lysis buffer (10 mM Tris-HCl pH 7.5, 150 mM NaCl, 0.5 mM EDTA, and 0.5% NP-40) containing phosphatase inhibitor cocktail 2/3 and protease inhibitor cocktail, and subjected to immunoprecipitation using GFP-trap beads (gtma; Chromotek). The immunoprecipitates were washed three times with wash buffer (10 mM Tris-HCl, pH 7.5, 150 mM NaCl, 0.5 mM EDTA, and 0.05% NP-40) and subjected to immunoblotting. The extraction of TX-100 soluble and insoluble fraction was performed as previously described.^108^ Briefly, the TX-100 soluble fraction from cells were prepared in TX-100 lysis buffer (0.25 mol/L sucrose, 10 mmol/L HEPES, pH 7.5, 1% TX-100, 150 mmol/L NaCl, and freshly added 1 mmol/L DTT) containing phosphatase inhibitor cocktail 2/3 and protease inhibitor cocktail. The cells in the lysis buffer were vortexed and incubated on ice for 15 min. The lysates were centrifuged at 14000 rpm for 10 min at 4°C. The supernatants were carefully collected without disrupting the pellet and used as TX-100-soluble fraction. Resultant pellets were washed twice in the lysis buffer and reconstituted in the lysis buffer containing 2% SDS, followed by sonication until the pellet is fully dispersed. Lysates were centrifuged at 14000 rpm for 5 min at 4°C and supernatants were collected as TX-100-insoluble fraction.

#### HaloTag-GFP and HaloTag-LC3 reporter processing assays

HaloTag-GFP and HaloTag-LC3 reporter processing assays was performed as previously described.^58^ Briefly, cells stably expressing HT-LC3 or HT-GFP were preincubated in complete medium containing 200 nM MPL for 20 min. The cells were then rinsed three times with DPBS, starved in the presence or absence of 100 nM BafA1 for 6 h, and analyzed by immunoblotting.

#### HaloTag-LC3 autophagosome completion assay and immunofluorescence

Cells were grown overnight on Lab-TekII Chambered Coverglass, Chamber Slide (154941; Nunc). For HT-LC3 autophagosome completion assay, cells were incubated in 13 MAS buffer (220 mM mannitol, 70 mM sucrose, 10 mM KH2PO4, 5 mM MgCl2, 2 mM HEPES, and 1 mM EGTA) containing 3 nM XF-PMP and 1 μM MIL at 37°C for 15 min, fixed in 4% PFA for 7 min, incubated in 13 MAS buffer containing 5 μM membrane permeable MPL for 30 min as described previously.^6^ For immunofluorescence, cells were fixed in 4% PFA for 7 min, permeabilized with 100 μg/mL digitonin for 7 min, blocked in 10% normal goat serum for 1 h, and incubated with primary antibodies overnight at 4°C followed by secondary antibodies for 1 h at room temperature (RT). Fluorescent images were obtained using a Leica AOBS SP8 laser-scanning confocal microscope (633 oil-immersion [1.2 numerical aperture] lens) with the highly sensitive HyD detectors and the Leica Application Suite X (LAS X), deconvolved using Huygens deconvolution software (Scientific Volume Imaging), and analyzed using Imaris software (Bitplane) and Volocity software (PerkinElmer) without gamma adjustment.

#### Histology and immunohistology

Tissue samples were fixed in 10% Neutral buffered formalin for 48 h, paraffin-embedded, sectioned at 5 μm thickness, deparaffinized and stained with hematoxylin and eosin (H&E). The H&E-stained tissues were blindly evaluated by a board-certified veterinary pathologist to assess the degree of severity of glial cell infiltration, which was scored in a routine semiquantitative manner^109–111^ from unremarkable (0), minimal (1), mild (2), to moderate (3). For liver immunohistology, deparaffinized sections were permeabilized with 100 μg/mL digitonin for 7 min at RT, blocked in 10% normal goat serum for 1 h, incubated with primary antibodies overnight at 4°C followed by secondary antibodies for 1 h at RT, treated with TrueVIEW autofluorescence quenching kit (SP-8400–15; Vector Laboratories), and analyzed by confocal microscopy as described above. For brain immunohistology, prenatal embryos were harvested and fixed in 4% (w/v) PFA overnight after pregnant mothers were euthanized with CO_2_ inhalation. Postnatal pups were anesthetized with ketamine (100 mg/kg)/xylazine (10 mg/kg) and were transcardially perfused with PBS followed by 4% PFA in PBS. The brains were removed and post-fixed in the same fixative at 4°C overnight. The fixed brains were cut into 40 μm slices with a vibratome, or cryopreserved in 30% sucrose (w/v) in PBS, embedded in the optimal cutting temperature compound (4583; Sakura Finetek USA) and sectioned at 20 μm thickness using a cryostat. The sections were pretreated for 30 min in 25 mM HCl at 65°C and rinsed with 40 mM borate buffer (pH 8.5), PBS, and TBS-T (10 mM Tris-HCl [pH 7.4], 100 mM NaCl with 0.3% Triton X-100 [v/v]). The sections were then blocked with a blocking buffer (5% normal donkey serum [v/v] in TBS-T) at 20°C–25°C for 1 h and incubated with primary antibodies diluted in blocking buffer overnight at 4°C. Sections were washed with TBS-T and then incubated with secondary antibodies with 4′6-diamino-2-phenylindole dihydrochloride (DAPI; D1306; Thermo Fisher Scientific) for nuclear staining for 1 h, and mounted with a Fluoro-Gel mounting medium (17985–11; Electron Microscopy Sciences). Images were captured with a Zeiss Axio Imager.M2 microscope using a Plan Apochromat 20x (NA 0.8) and the Axiocam 305 CCD camera (Carl Zeiss AG; Jena, Germany).

#### Electron microscopy

For cell electron microscopy, cells were grown on the Permanox dishes (174888; Thermo Fisher Scientific) overnight, starved in starvation medium for 3 h, fixed in 2.5% glutaraldehyde and 2% paraformaldehyde in 0.1 M phosphate buffer (pH 7.4) for 1.5 h at RT followed by post fixation buffer (1% osmium tetroxide/1.5% potassium ferrocyanide/0.1 M phosphate buffer (pH 7.4) for 1 h. For liver electron microscopy, postnatal pups were anesthetized with ketamine (100 mg/kg)/xylazine (10 mg/kg) and were transcardially perfused with PBS followed by 4% PFA in PBS. The livers were removed and fixed in 2.5% glutaraldehyde and 2% paraformaldehyde in 0.1 M phosphate buffer (pH 7.4) for 1.5 h at RT followed by post fixation buffer (1% osmium tetroxide/1.5% potassium ferrocyanide/0.1 M phosphate buffer (pH 7.4) overnight at 4°C. After post fixation, cell and liver samples were dehydrated in a graded series of ethanol and acetone, embedded in LX-112 (Ladd Research, Williston, VT), sectioned at a thickness of 65 nm, mounted on the grids, stained with aqueous uranyl acetate and lead citrate, and analyzed using a JEOL JEM1400 Transmission Electron Microscope (JEOL USA Inc., Peabody, MA, USA) located at the Penn State College of Medicine Transmission Electron Microscopy Core.

#### EGFR degradation assay

EGFR degradation assay was performed as previously described.^112^ Briefly, cells at 70–80% confluency were incubated in DMEM containing 0.5% FBS for 4 h followed by addition of 10 μg/mL CHX for 20 min to prevent *de novo* protein synthesis. The cells were then treated with 100 ng/mL EGF in the presence of 10 μg/mL CHX for the indicated periods of time and subjected to immunoblotting.

#### Plasma cytokine and ALT assay

Plasma cytokine levels were measured using semiquantitative Mouse Cytokine Array C2000 (AAM-CYT-2000; RayBio) according to the manufacturer’s instruction. Signals were imaged using ChemiDoc MP imaging system (Bio-Rad) and spot intensity were analyzed using ImageJ^105^ with plug-in “Protein array analyzer” (2010; http://rsb.info.nih.gov/ij/macros/toolsets/Protein%20Array%20Analyzer.txt) made by Carpentier G. Plasma ALT levels were measured using Mouse ALT ELISA Kit (ab282882; Abcam) according to the manufacturer’s instruction.

#### RNA-seq and qPCR

Liver samples isolated from 8-wk-old mice were immediately immersed in RNAlater stabilization solution (AM7020; Invitrogen) and subjected to total RNA extraction using RNeasy Plus Kit (74134, QIAGEN). mRNA-seq was performed by Novogene Corporation, Inc. Briefly, total RNA samples were quality checked by NanoDrop and Agilent Bioanalyzer 2100. cDNA libraries were generated with NEBNext Ultra II Directional RNA Library Prep Kit for Illumina (E7760, New England Biolobs). The resultant libraries were checked using Qubit and real-time PCR for quantification and Agilent bioanalyzer for size distribution detection. Quantified libraries were pooled and sequenced on Illumina Novaseq 6000 platform using PE150 reads, according to effective library concentration and data amount. Raw reads of FASTQ format were processed through the FASTQ software and clean reads were obtained by trimming reads containing adapter, reads containing ploy-N and low-quality reads. The trimmed FASTQ files were mapped against the mm10 mouse reference genome using Hisat2 v2.0.5. featureCounts v1.5.0-p3 and DESeq2 were used to count the reads numbers mapped to each gene and normalize gene count data, respectively. Differential expression gene and gene set enrichment analyses were performed using the integrated differential expression and pathway analysis (iDEP) platform (v0.96). Real-time PCR amplification was performed using the iTaq Universal SYBR Green Supermix (1725121, Bio-rad) and the primer sets listed in Table S3. Fluorescence was monitored using the CFX96 Touch Real-Time PCR Detection System (Bio-rad). Results were analyzed using the ΔΔCT method and normalized to β-glucuronidase.

#### APEX2 proximity proteomics

Cells expressing Apex2 fusion proteins were starved for 90 min and incubated with 500 μM biotin-phenol (LS-3500.0250, Iris Biotech) at 37°C for 30 min followed by 1 mM H_2_O_2_ treatment at RT for 1 min. The cells were then washed three times with a quencher solution (5 mM Trolox, 10 mM sodium ascorbate, and 10 mM sodium azide), harvested and lysed in RIPA buffer supplemented with 5 mM Trolox, 10 mM sodium ascorbate, 10 mM sodium azide, and the protease inhibitor cOmplete mini. To enrich biotinylated proteins, an equal amount of cell lysates was subjected to affinity purification using Dynabeads MyOne Streptavidin C1 (65001; Thermo Fisher Scientific) overnight at 4°C. Beads were subsequently washed as follows: twice with RIPA buffer, once with 2% SDS, once with 1 M KCl, once with 0.1 M NaCO3, twice with RIPA buffer, twice with 10 mM Tris/Cl pH 7.5, 150 mM NaCl, 0.5 mM EDTA, 0.05% NP40, and twice with 50 mM Tris, 50 mM NaCl, followed by resuspension in 50 mM NH_4_HCO_3_. For proteomics, protein bound on beads were washed three times with 0.5 M tetraethylammonium bicarbonate (TEAB), reduced and alkylated with Tris(2-carboxyethyl) phosphine (TCEP) buffer (10 mM TCEP, 0.5 M TEAB, 20 mM chloroacetamide, 2% sodium deoxycholate, pH 8.5) at 80°C for 10 min, and digested with 0.5 μg of Trypsin LysC (Promega) at 30°C overnight. Peptides were purified using CDS Empore SDB-RPS tips, and run on a Bruker TIMS TOF Flex in-line with a nanoflow Bruker Fifteen column (15 cm × 150 μm ID, 1.5 μm C18, 120Å) using a NanoElute LC with a gradient consisting of 5%–30% (ACN, 0.1% FA) over 17.22 min at 1 μL/min using the DDA PASEF short analysis method at the Penn State College of Medicine Mass Spectrometry and Proteomics. The raw data were analyzed using Fragpipe v.19.0 and MSFragger v.3.7 software^106^ with a Human database UP000005640, including 536 common lab contaminant proteins, decoys, and a total of 196514 entries. Precursor mass tolerance was set at 20 ppm, Fragment mass tolerance at 20 ppm. Carbamidomethylation of cysteine residues (+57.021 Da) was applied as a static modification, and oxidation of methionine residues (+15.995 Da), and phosphorylations of tyrosine, threonine, and serine residues (+79.9663 Da) were considered as variable modifications. Peptide-spectrum matches (PSMs) were adjusted to 1%. MS data were processed and analyzed using Scaffold 5.3.0 Proteome software. The total precursor intensity data was normalized and imputed for analyses as described previously.^113^

### QUANTIFICATION AND STATISTICAL ANALYSIS

Statistical significance was determined using Graph Pad Prism 7.0. Threshold for statistical significance for each test was set at 95% confidence (*p* < 0.05). Statistical details for individual experiments are provided in the corresponding figure legends.

## Supplementary Material

1

2

3

4

5

6

7

## Figures and Tables

**Table:** KEY RESOURCES TABLE

REAGENT or RESOURCE	SOURCE	IDENTIFIER
Antibodies		

Mouse monoclonal α-tubulin	Sigma-Aldrich	Cat# T5168; RRID:AB_477579
Mouse monoclonal β-actin	Sigma-Aldrich	Cat# A5441; RRID:AB_476744
Mouse monoclonal FLAG	Sigma-Aldrich	Cat# F1804; RRID:AB_262044
Mouse monoclonal IκBα	Cell Signaling Technology	Cat# 4814; RRID:AB_390781
Mouse monoclonal LC3B	MBL International	Cat# M152–3; RRID:AB_1279144
Mouse monoclonal TSG101	GeneTex	Cat# GTX70255; RRID:AB_373239)
Mouse monoclonal Ubiquitin	Novus	Cat# NB300–130; RRID:AB_2238516
Mouse monoclonal VPS28	Santa Cruz Biotechnology	Cat# sc-166537; RRID:AB_2214880
Rabbit monoclonal Atg5	Cell Signaling Technology	Cat# 12994; RRID:AB_2630393
Rabbit monoclonal Atg7	Cell Signaling Technology	Cat# 8558; RRID:AB_10831194
Rabbit monoclonal Atg13	Cell Signaling Technology	Cat# 13273; RRID:AB_2798169
Rabbit monoclonal EEA1	Thermo Fisher Scientific	Cat# MA5–14794; RRID:AB_10985824
Rabbit monoclonal EGFR	Cell Signaling Technology	Cat# 4267; RRID:AB_2246311
Rabbit monoclonal GFP	Cell Signaling Technology	Cat# 2956; RRID:AB_1196615)
Rabbit polyclonal Gstm1	Proteintech	Cat# 12412–1-AP; RRID:AB_2115925
Rabbit polyclonal HaloTag	Promega	Cat# G9281; RRID:AB_713650
Rabbit polyclonal Iba1	FUJIFILM Wako Pure Chemical Corporation	Cat# 019–19741; RRID:AB_839504
Rabbit polyclonal Keap1	Proteintech	Cat# 10503–2-AP; RRID:AB_2132625
Rabbit polyclonal MAP1LC3B	Novus	Cat# NB100–2220; RRID:AB_10003146
Rabbit Monoclonal MGST3	Abcam	Cat#ab192254
Rabbit polyclonal MVB12A	Fisher scientific	Cat#50–173-5082
Rabbit polyclonal NQO1	Proteintech	Cat# 11451–1-AP; RRID:AB_2298729
Rabbit polyclonal NRF2	Cell Signaling Technology	Cat# 12721; RRID:AB_2715528
Rabbit Monoclonal phospho-Atg13 Ser355	Cell Signaling Technology	Cat# 46329; RRID:AB_3064843
Rabbit polyclonal -p62 Ser349	Cell Signaling Technology	Cat# 95697; RRID:AB_2800251
Rabbit Monoclonal phospho-p62 Ser403	Cell Signaling Technology	Cat# 39786; RRID:AB_2799162
Rabbit Monoclonal phospho-TBK1	Cell Signaling Technology	Cat# 5483; RRID:AB_10693472
Rabbit polyclonal UBAP1	Proteintech	Cat# 12385–1-AP; RRID:AB_2211886
Rabbit polyclonal VPS37A	Sigma-Aldrich	Cat# HPA024705; RRID:AB_10601776
Rabbit polyclonal VPS37A	Proteintech	Cat# 11870–1-AP; RRID:AB_2215230
Chicken polyclonal GFAP	EnCor Biotechnology	Cat# CPCA-GFAP; RRID:AB_2109953
Guinea pig polyclonal p62	ARP American Research Products	Cat# 03-GP62-C; RRID:AB_1542690
Rat monoclonal CD11b	Bio-Rad	Cat# MCA711; RRID:AB_321292
Rat monoclonal LAMP1	Santa Cruz Biotechnology	Cat# sc-19992; RRID:AB_2134495
Alexa Fluor^™^ 488-conjugated goat antibodies against mouse IgG	Thermo Fisher Scientific	Cat# A28175; RRID:AB_2536161
Alexa Fluor^™^ 488-conjugated goat antibodies against rabbit IgG	Thermo Fisher Scientific	Cat# A-11008; RRID:AB_143165
Alexa Fluor 568-conjugated goat antibodies against rabbit IgG	Thermo Fisher Scientific	Cat# A-11036; RRID:AB_10563566
Alexa Fluor 647-conjugated goat antibodies against rat IgG	Life Technologies	Cat#A21247
HRP-conjugated goat antibodies against rabbit IgG	Cell Signaling Technology	Cat# 7074; RRID:AB_2099233
IRDye 680RD-conjugated donkey antibodies against mouse IgG	LI-COR Biosciences	Cat# 926–68072; RRID:AB_10953628
IRDye 680RD-conjugated donkey antibodies against guinea pig IgG	LI-COR Biosciences	Cat# 926–68077; RRID:AB_10956079
IRDye 800CW-conjugated donkey antibodies against rabbit IgG	LI-COR Biosciences	Cat# 926–32213; RRID:AB_621848

Chemicals; peptides; and recombinant proteins		

Dulbecco’s Modification of Eagle’s Medium (DMEM)	Corning	Cat# 10–013-CV
Amino acid-free DMEM	FUJIFILM Wako Pure Chemical Corporation	Cat# 048–33575
McCoy’s 5A medium	Corning	Cat# 10–050-CV
Antibiotic antimycotic solution	Cytiva	Cat# SV30079.01
5Z-7-oxozeaenol	MedChemExpress	Cat# HY-12686
AlexaFluor 488-conjugated membrane-impermeable ligand (MIL)	Promega	Cat# G1001
Bafilomycin A1	Thermo Fisher Scientific	Cat# AAJ61835MCR
Cycloheximide	Sigma-Aldrich	Cat# C7698
Digitonin	Sigma-Aldrich	Cat# D141
EGF from murine submaxillary gland	Sigma-Aldrich	Cat# E4127
GFP-trap magnetic agarose	Chromotek	Cat# gtma
GSK8612	MedChemExpress	Cat# HY-111941
Mouse recombinant TNFα	Prospec	Cat# CYT-252
Normal goat serum	Sigma-Aldrich	Cat# G9023
Paraformaldehyde	Electron Microscopy Sciences	Cat# 15710
Phosphatase inhibitor cocktail 2	Sigma-Aldrich	Cat# P5726
Phosphatase inhibitor cocktail 2	Sigma-Aldrich	Cat# P0044
Protease inhibitor cocktail	Sigma-Aldrich	Cat# P8340
Protease inhibitor cOmplete mini	Sigma-Aldrich	Cat# 11836153001
SBI-0206965	Xcess biosciences	Cat# M60268
Tetramethylrhodamine-conjugated membrane-permeable ligand (MPL)	Promega	Cat# G8251
XF Plasma Membrane Permeabilizer (XF-PMP)	Agilent	Cat# 102504–100

Critical commercial assays		

Mouse ALT ELISA Kit	Abcam	Cat# ab282882
Mouse Cytokine Array C2000	RayBio	Cat# AAM-CYT-2000

Deposited data		

Mass spectrometry data	This study	PRIDE: PXD057301

Experimental models: Cell lines		

Human: HEK293T/17	ATCC	Cat# CRL-11268; RRID:CVCL_1926
Human: HuH-7	Dr. Jianming Hu	RRID:CVCL_0336
Human: Hep G2	ATCC	Cat# HB-8065; RRID:CVCL_0027
Human: Phoenix-AMPHO	Dr. Garry Nolan	RRID:CVCL_H716
Human: U-2 OS	ATCC	Cat# HTB-96; RRID:CVCL_0042

Experimental models: Organisms/strains		

Mouse: B6.Cg-Edil3Tg(Sox2-cre)1Amc/J	The Jackson Laboratory	Cat# JAX 008454; RRID:IMSR_JAX:008454
Mouse: C57BL/6J	The Jackson Laboratory	Cat# JAX 000664; RRID: RRID:IMSR_JAX:000664
Mouse: C57BL/6 VPS37A^flox/flox^	Cyagen	N/A

Oligonucleotides		

Primers	See Tables S3 and S4	–
siRNA ON-TARGETplus SMART Pool Non-targeting	Dharmacon	Cat# D-001810-10
siRNA ON-TARGETplus SMART Pool mouse TBK1	Dharmacon	Cat# L-063162-00

Recombinant DNA		

Plasmid: FUGW-PK-hLC3	Tanida et al.^100^	Addgene Plasmid #61460; RRID:Addgene_61460
Plasmid: mVenus-C1	Koushik et al.^101^	Addgene Plasmid #27794; RRID:Addgene_27794
Plasmid: pBABE-neo largeT cDNA	Hahn et al.^102^	Addgene Plasmid #1780; RRID:Addgene_1780
Plasmid: pCDH-Cuo-MCS-EF1α-CymR-T2A-Puro	System Biosciences	Cat#QM800A-1;
Plasmid: pEGFP-flag-APEX2-α-tubulin	Lam et al.^70^	Addgene Plasmid #66171; RRID:Addgene_66171
Plasmid: pEGFP-VPS4-E228Q	Votteler et al.^103^	Addgene Plasmid #80351; RRID:Addgene_80351
Plasmid: pMRX-IB-HaloTag7-mGFP	Yim et al.^58^	Addgene Plasmid #184903; RRID:Addgene_184903
Plasmid: lentiCRISPRv2 puro	Stringer et al.^104^	Addgene Plasmid #98290; RRID:Addgene_98290
Plasmid: lentiCRISPR v2 targeting hVPS37A #1–3	Takahashi et al.^7^	N/A
Plasmid: lentiCRISPR v2 targeting mATG7	This study	N/A
Plasmid: pCDH1-Ubc-HT-LC3	This study	N/A
Plasmid: pCDH-mVenus-Hygro	This study	N/A
Plasmid: pCDH1-mVenus-hVPS37A WT	This study	N/A
Plasmid: pCDH1-mVenus-hVPS37A K382D	This study	N/A
Plasmid: pCDH1-mVenus-mVPS37A WT	This study	N/A
Plasmid: pCDH1-mVenus-mVPS37A K382D	This study	N/A
Plasmid: pCDH-FLAG-APEX2-LC3B	This study	N/A
Plasmid: pCDH-TRE3G-GFPDNVPS4-tet-on-3G	This study	N/A

Software and algorithms		

Image Studio software version 5.2	LI-COR Biosciences	RRID:SCR_015795
GraphPad Prism 7.0	GraphPad Prism	RRID:SCR_002798
Leica Application Suite X	Leica	RRID:SCR_013673
Huygens	Scientific Volume Imaging	RRID:SCR_014237
Volocity	PerkinElmer	RRID:SCR_002668
Imaris	Bitplane	RRID:SCR_007370
ImageJ	Schneider et al.^105^	RRID:SCR_003070
Fragpipe v.19.0	Nesvizhskii lab	RRID:SCR_022864
MSFragger v.3.7	Nesvizhskii lab^106^	N/A
Scaffold Proteome Software	Proteome Software, Inc.	RRID:SCR_014345

## Highlights

- Autophagosome closure-defective mice were generated by targeting VPS37A UEVL
- Homozygous mutant mice showed signs of autophagy impairment
- About half of the mutant neonates survived to adulthoodwithout hepatomegaly
- LC3-conjugated phagophores facilitate TBK1-dependent p62 phosphorylation and aggregation
