# Supplementary material for: Unveiling the physiological impact of ESCRT-dependent autophagosome closure by targeting the VPS37A ubiquitin E2 variant-like domain
Source: Cell Rep. Author manuscript; Available in PMC 2025 Jan 21. (PMC11748760; doi:10.1016/j.celrep.2024.115016)
Supplement: 1 [file NIHMS2044445-supplement-1.pdf]

Cell Reports, Volume *43*

Supplemental information

Unveiling the physiological impact of ESCRT-dependent autophagosome closure by targeting the VPS37A ubiquitin E2 variant-like domain

Kouta Hamamoto, Xinwen Liang, Ayako Ito, Matthew Lanza, Van Bui, Jiawen Zhang, David M. Opozda, Tatsuya Hattori, Longgui Chen, David Haddock, Fumiaki Imamura, Hong-Gang Wang, and Yoshinori Takahashi

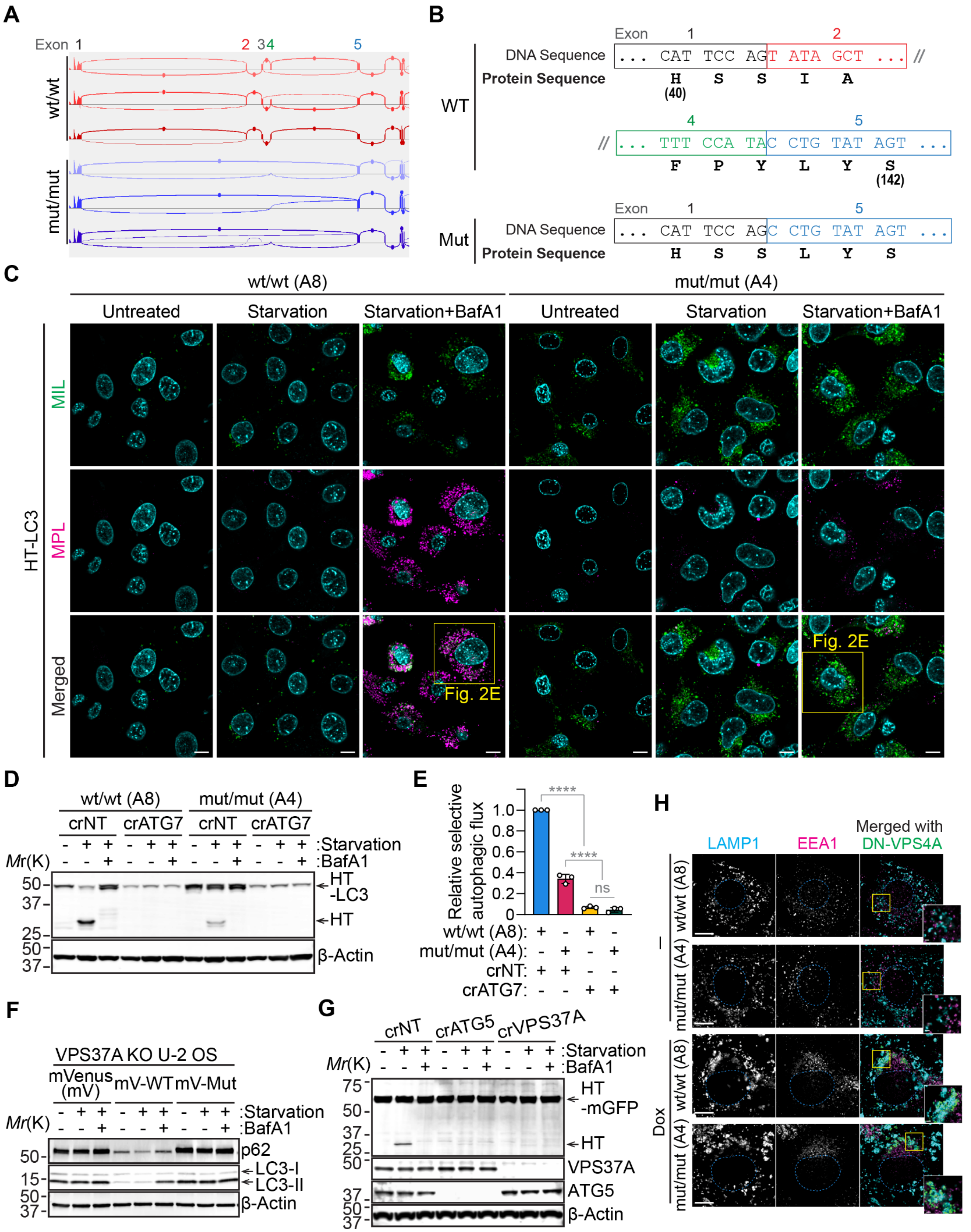
A
Exon 1
2 34
5
wt/wt
mut/mut
B
WT
Exon 1 2
DNA Sequence ... CAT TCC AGT ATA GCT ...
Protein Sequence H S S I A
(40)
4 5
... TTT CCA TAC CTG TAT AGT ...
F P Y L Y S
(142)
Mut
Exon 1 5
DNA Sequence ... CAT TCC AGC CTG TAT AGT ...
Protein Sequence H S S L Y S
C
wt/wt (A8)
mut/mut (A4)
Untreated
Starvation
Starvation+BafA1
HT-LC3
MIL
MPL
Merged
Fig. 2E
D
wt/wt (A8)
mut/mut (A4)
crNT
crATG7
:Starvation
:BafA1
Mr(K)
50
37
25
HT-LC3
HT
β-Actin
E
Relative selective autophagic flux
1.0
0.8
0.6
0.4
0.2
0
****
****
ns
wt/wt (A8): + - + -
mut/mut (A4): - + - +
crNT: + + - -
crATG7: - - + +
F
VPS37A KO U-2 OS
mVenus (mV)
mV-WT
mV-Mut
:Starvation
:BafA1
Mr(K)
p62
LC3-I
LC3-II
β-Actin
G
crNT
crATG5
crVPS37A
:Starvation
:BafA1
Mr(K)
75
50
37
25
HT-mGFP
HT
VPS37A
ATG5
β-Actin
H
LAMP1
EEA1
Merged with DN-VPS4A
wt/wt (A8)
mut/mut (A4)
Dox

Figure S1 The VPS37A ubiquitin E2 variant-like (UEVL) domain is uniquely required for autophagosome closure. (A and B) Sashimi plot (A) and sequencing (B) of VPS37A transcripts in the indicated MEFs. (C) Confocal images of HT-LC3-expressing immortalized MEFs that were starved in the presence or absence of 100 nM BafA1 for 3 h and subjected to the HaloTag-LC3 assay. MIL, Alexa Fluor 488-conjugated membrane-impermeable HT ligand; MPL, tetramethylrhodamine (TMR)-conjugated membrane-permeable HT ligand. Magnified images in the indicated areas are shown in Fig. 2E. Scale bars: 10 µm. (D) Immunoblot analysis of HT-LC3-expressing immortalized MEFs that were pulse-labeled with MPL for 20 min, and starved for 6 h. (E) Bar plot of HT/(HT-LC3 + HT) ratio relative to wt/wt MEFs in (D) (n = 3). Statistical significance was determined by one-way ANOVA followed by Tukey's multiple comparison test. All values are mean ± SD. ****, $p \leq 0.0001$; ns, not significant. (F) Immunoblot analysis of VPS37A KO U-2 OS cells stably expressing mVenus (mV) or mV-VPS37A WT (WT) or mV-VPS37A ΔUEVL (Mut) incubated in complete medium or starvation medium in the presence or absence of Bafilomycin A1 (BafA1) for 3h. (G) Immunoblot analysis of U-2 OS cells with the HT-mGFP bulk autophagic flux reporter, pulse-labeled for 20 min with MPL, and starved for 6 h in the presence or absence of BafA1. (H) Confocal images of MEFs transduced with lentiviruses encoding doxycycline (Dox)-inducible GFP-VPS4A (E228Q) (DN-VPS4A), pre-incubated in complete medium in the presence or absence of 1 µg/ml Dox, stained with indicated antibodies. Scale bars: 10 µm; 1 µm in magnified images.

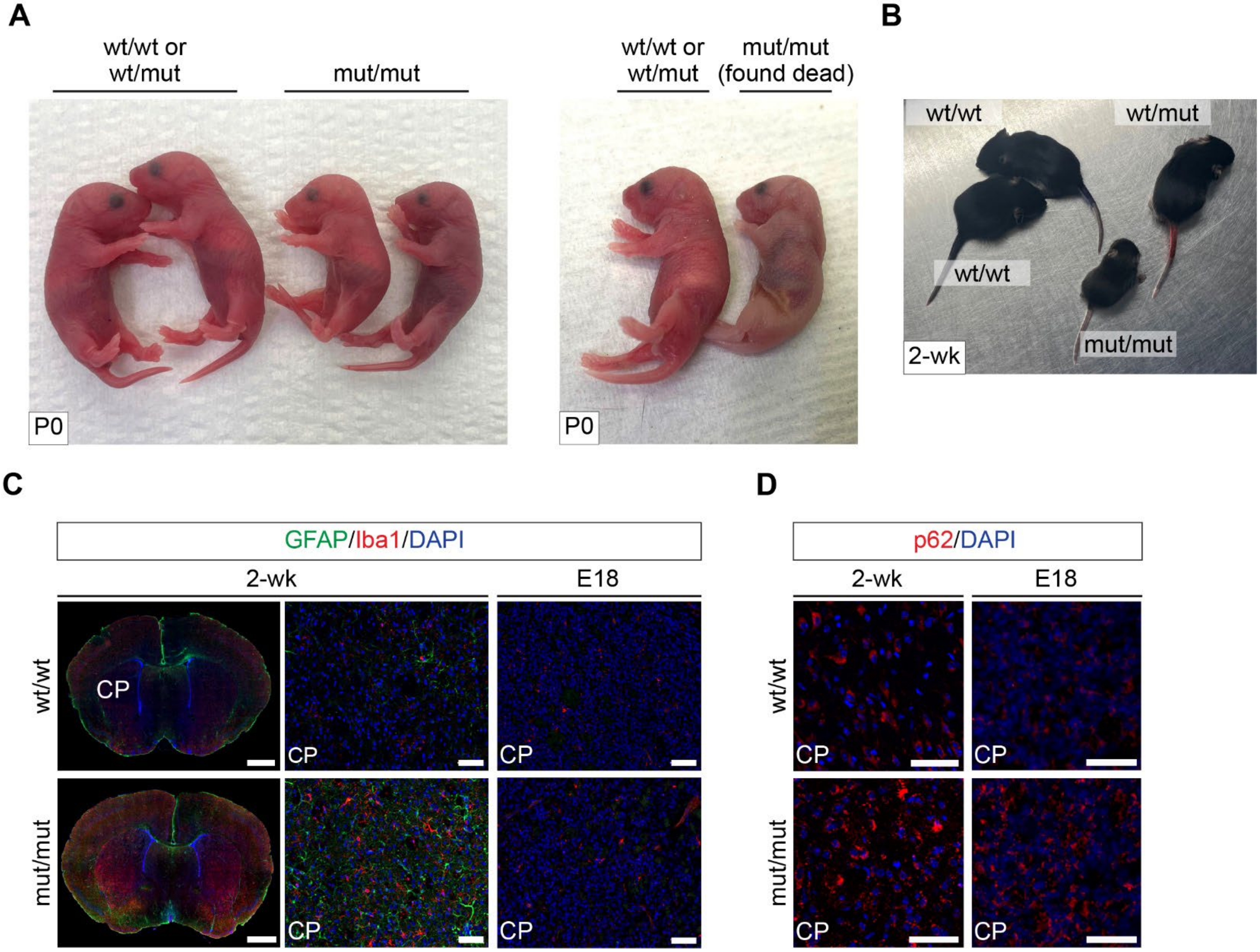

Figure S2. Homozygous VPS37A UEVL mutant mice are born smaller than their wild-type and heterozygous littermates and display growth retardation along with neuroinflammation.

(A and B) Images of newborn (P0) (A) and 2-wk-old (B) mice with the indicated genotypes. (C and D) Fluorescence images of coronal sections of 2-wk-old and E18 mouse brains stained with the indicated antibodies. Nuclei were counterstained with DAPI. CP, caudate putamen. Scale bars: 1 mm in whole brain; 50 μm in CP.

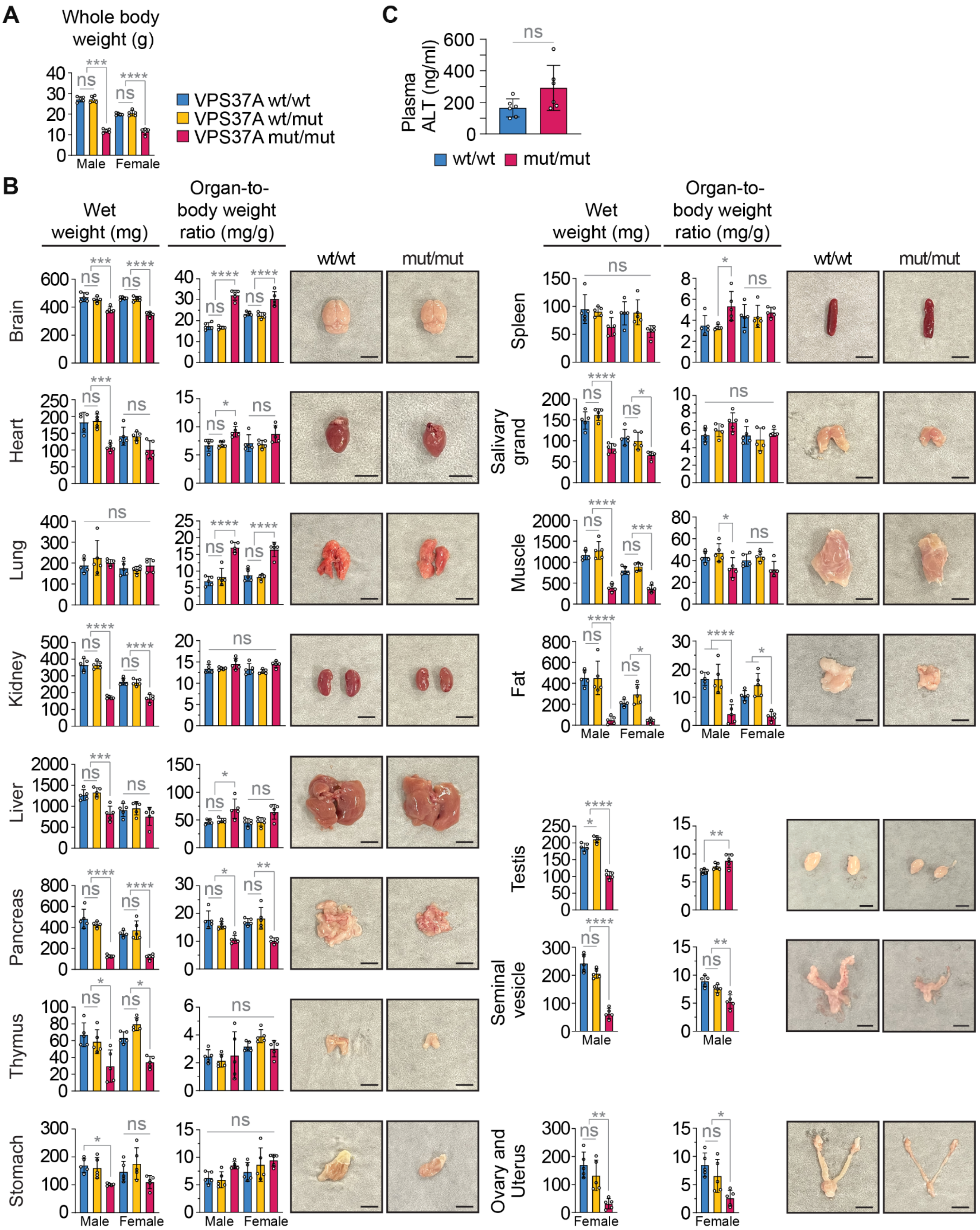
A
Whole body weight (g)
Male
Female
VPS37A wt/wt
VPS37A wt/mut
VPS37A mut/mut
C
Plasma ALT (ng/ml)
wt/wt
mut/mut
B
Wet weight (mg)
Organ-to-body weight ratio (mg/g)
wt/wt
mut/mut
Brain
Heart
Lung
Kidney
Liver
Pancreas
Thymus
Stomach
Spleen
Salivary grand
Muscle
Fat
Testis
Seminal vesicle
Ovary and Uterus
Male
Female

Figure S3. Body and tissue weight, organ-to-body weight ratios and gross tissue images of 8-wk-old wild-type, heterozygous, and homozygous VPS37A mutant mice. (A and B) Bar graphs of whole-body weight (g) (A), wet tissue weight (mg) and organ-to-body weight ratio (B) of 8-wk-old mice ($n = 5$ each of male and female mice). In (B), representative tissue images are shown on the right. Statistical significance was determined by one-way ANOVA followed by Tukey's multiple comparison test. Scale bars: 5 mm. (C) Bar graphs of alanine aminotransferase (ALT) levels in plasma from 8-wk-old mice ($n = 5$). Statistical significance was determined by Student's t-test. All values in the bar graphs are mean ± SD. *$p \leq 0.05$; **$p \leq 0.01$; ***$p \leq 0.001$; ****$p \leq 0.0001$; ns, not significant.

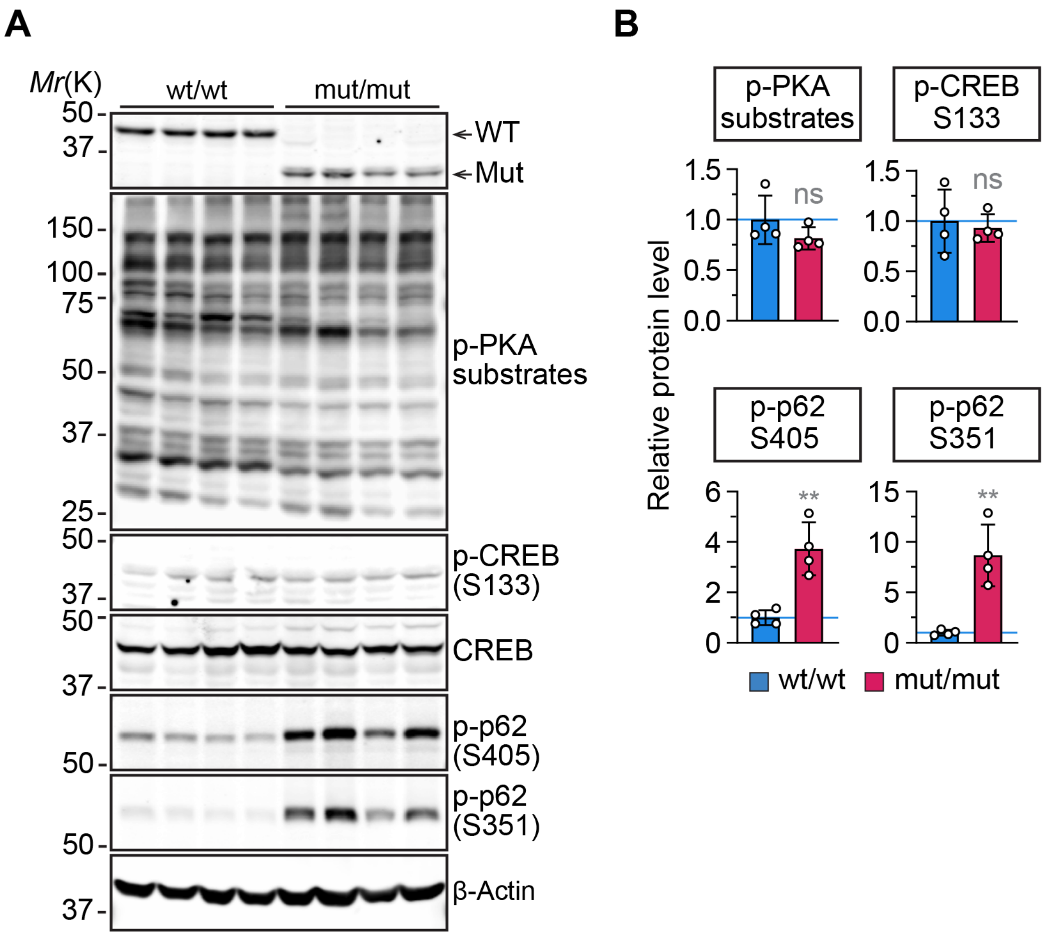

Figure S4. VPS37A UEVL mutant has minimal impact on glucagon receptor signaling. (A) Immunoblot analyses of liver homogenates prepared from 8-wk-old mice. (B) Bar plots of the protein levels relative to wt/wt livers in (A) (n = 4). Statistical significance was determined by Welch's t-test. All values in the bar graphs are mean ± SD. **$p \leq 0.01$; ns, not significant.

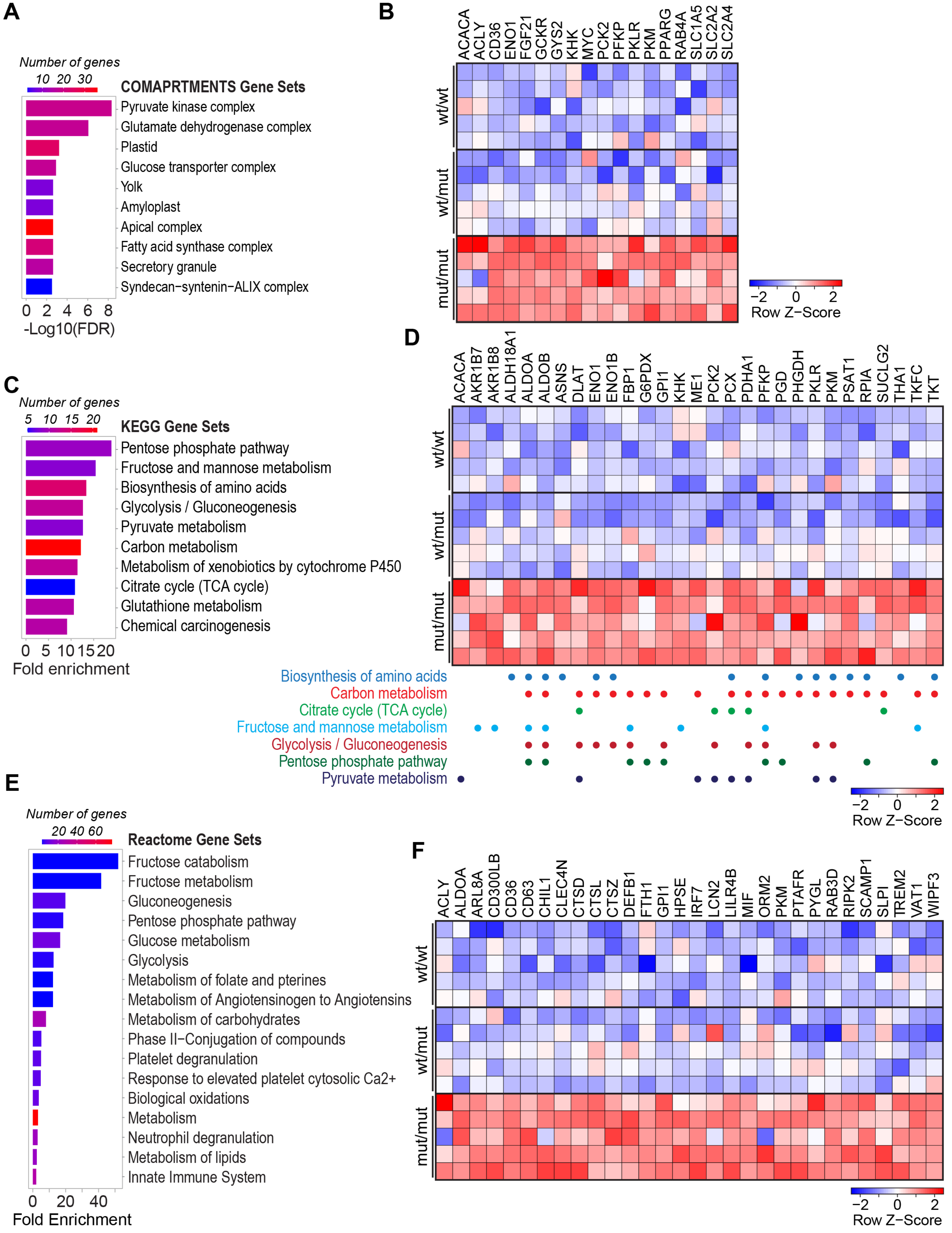
A
Number of genes
10 20 30
COMAPRTMENTS Gene Sets
Pyruvate kinase complex
Glutamate dehydrogenase complex
Plastid
Glucose transporter complex
Yolk
Amyloplast
Apical complex
Fatty acid synthase complex
Secretory granule
Syndecan−syntenin−ALIX complex
0 2 4 6 8
-Log10(FDR)
B
ACACA ACLY CD36 ENO1 FGF21 GCKR GYS2 KHK MYC PCK2 PFKP PKLR PKM PPARG RAB4A SLC1A5 SLC2A2 SLC2A4
wt/wt
wt/mut
mut/mut
−2 0 2
Row Z−Score
C
Number of genes
5 10 15 20
KEGG Gene Sets
Pentose phosphate pathway
Fructose and mannose metabolism
Biosynthesis of amino acids
Glycolysis / Gluconeogenesis
Pyruvate metabolism
Carbon metabolism
Metabolism of xenobiotics by cytochrome P450
Citrate cycle (TCA cycle)
Glutathione metabolism
Chemical carcinogenesis
0 5 10 15 20
Fold enrichment
D
ACACA AKR1B7 AKR1B8 ALDH18A1 ALDOA ALDOB ASNS DLAT ENO1 ENO1B FBP1 G6PDX GPI1 KHK ME1 PCK2 PCX PDHA1 PFKP PGD PHGDH PKLR PKM PSAT1 RPIA SUCLG2 THA1 TKFC TKT
wt/wt
wt/mut
mut/mut
Biosynthesis of amino acids
Carbon metabolism
Citrate cycle (TCA cycle)
Fructose and mannose metabolism
Glycolysis / Gluconeogenesis
Pentose phosphate pathway
Pyruvate metabolism
−2 0 2
Row Z−Score
E
Number of genes
20 40 60
Reactome Gene Sets
Fructose catabolism
Fructose metabolism
Gluconeogenesis
Pentose phosphate pathway
Glucose metabolism
Glycolysis
Metabolism of folate and pterines
Metabolism of Angiotensinogen to Angiotensins
Metabolism of carbohydrates
Phase II−Conjugation of compounds
Platelet degranulation
Response to elevated platelet cytosolic Ca2+
Biological oxidations
Metabolism
Neutrophil degranulation
Metabolism of lipids
Innate Immune System
0 20 40
Fold Enrichment
F
ACLY ALDOA ARL8A CD300LB CD36 CD63 CHIL1 CLEC4N CTSD CTSL CTSZ DEFB1 FTH1 GPI1 HPSE IRF7 LCN2 LILR4B MIF ORM2 PKM PTAFR PYGL RAB3D RIPK2 SCAMP1 SLPI TREM2 VAT1 WIPF3
wt/wt
wt/mut
mut/mut
−2 0 2
Row Z−Score

Figure S5. VPS37A UEVL loss upregulates glucose transporter and carbohydrate metabolism regulatory genes in the liver. (A, C and E) Bar plots of top 10 Jensen cellular COMPARTMENTS (A), KEGG biological pathways (C) and Reactome pathways (E) enriched by homozygous VPS37A UEVL mutation in the liver (FDR < 0.05). (B, D and F) Heatmaps of the glucose transporter complex regulatory genes (B), carbohydrate metabolism regulatory genes (D), and innate immune system pathway genes (F) identified in (A), (C), and (E), respectively.

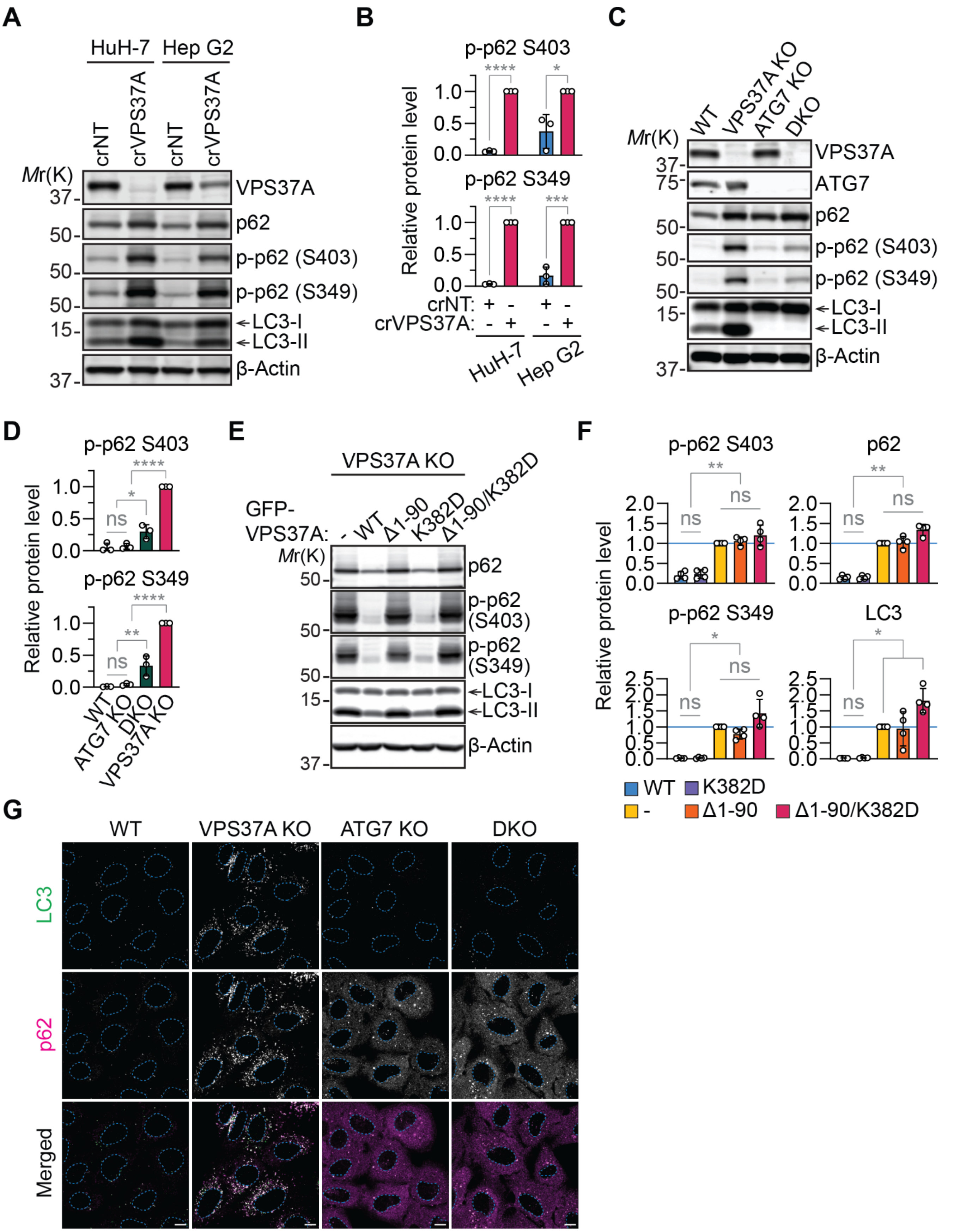

Figure S6. VPS37A UEVL loss promotes p62 phosphorylation and large p62 aggregate formation in an ATG7-dependent manner. (A) Immunoblot analysis of the indicated HuH-7 and Hep G2 cells. (B) Bar plots of

phosphorylated p62 levels relative to crVPS37A-transduced cells in (A) (n = 3). (C) Immunoblot analysis of U-2 OS cells with the indicated genotypes. (D) Bar plots of phosphorylated p62 levels relative to VPS37A KO cells in (C) (n = 3). (E) Immunoblot analysis of VPS37A KO U-2 OS cells expressing the indicated plasmids. (F) Bar plots of the indicated protein levels relative to crVPS37A-transduced cells in (E) (n = 4). (G) Confocal images of U-2 OS cells stained for p62 and LC3. Scale bars: 10 μm. Statistical significance was determined by Student's t-test (B), and one-way ANOVA followed by Tukey's multiple comparison test (D, F). All values in the graphs are mean ± SD. *$p \leq 0.05$; **$p \leq 0.01$; ***$p \leq 0.001$; ****$p \leq 0.0001$; ns, not significant.

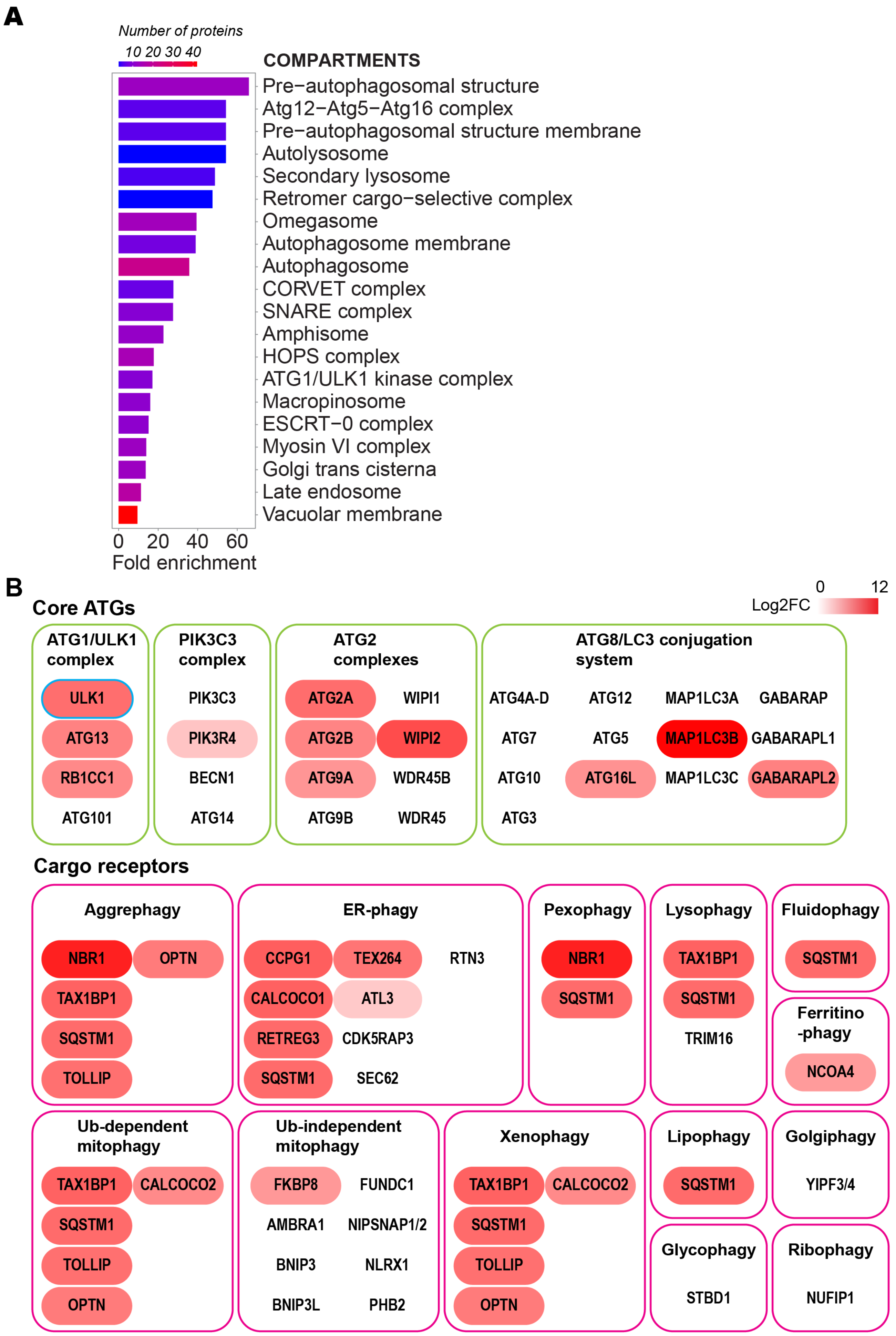
A
Number of proteins
10 20 30 40
COMPARTMENTS
Pre−autophagosomal structure
Atg12−Atg5−Atg16 complex
Pre−autophagosomal structure membrane
Autolysosome
Secondary lysosome
Retromer cargo−selective complex
Omegasome
Autophagosome membrane
Autophagosome
CORVET complex
SNARE complex
Amphisome
HOPS complex
ATG1/ULK1 kinase complex
Macropinosome
ESCRT−0 complex
Myosin VI complex
Golgi trans cisterna
Late endosome
Vacuolar membrane
0 20 40 60
Fold enrichment
B
Core ATGs
0 12
Log2FC
ATG1/ULK1 complex
ULK1
ATG13
RB1CC1
ATG101
PIK3C3 complex
PIK3C3
PIK3R4
BECN1
ATG14
ATG2 complexes
ATG2A
WIPI1
ATG2B
WIPI2
ATG9A
WDR45B
ATG9B
WDR45
ATG8/LC3 conjugation system
ATG4A-D
ATG12
MAP1LC3A
GABARAP
ATG7
ATG5
MAP1LC3B
GABARAPL1
ATG10
ATG16L
MAP1LC3C
GABARAPL2
ATG3
Cargo receptors
Aggrephagy
NBR1
OPTN
TAX1BP1
SQSTM1
TOLLIP
ER-phagy
CCPG1
TEX264
RTN3
CALCOCO1
ATL3
RETREG3
CDK5RAP3
SQSTM1
SEC62
Pexophagy
NBR1
SQSTM1
Lysophagy
TAX1BP1
SQSTM1
TRIM16
Fluidophagy
SQSTM1
Ferritino -phagy
NCOA4
Ub-dependent mitophagy
TAX1BP1
CALCOCO2
SQSTM1
TOLLIP
OPTN
Ub-independent mitophagy
FKBP8
FUNDC1
AMBRA1
NIPSNAP1/2
BNIP3
NLRX1
BNIP3L
PHB2
Xenophagy
TAX1BP1
CALCOCO2
SQSTM1
TOLLIP
OPTN
Lipophagy
SQSTM1
Golgiphagy
YIPF3/4
Glycophagy
STBD1
Ribophagy
NUFIP1

Figure S7. Autophagy regulatory proteins enriched by phagophore-conjugated APEX2-LC3. Bar plot of top 20 Jensen cellular COMPARTMENTS (A), and heat diagram of core ATG proteins and autophagic cargo receptors (B) enriched with the 187 phagophore-associated proteins identified in Fig. 7B (FDR < 0.05). In (B), enriched proteins are colored.

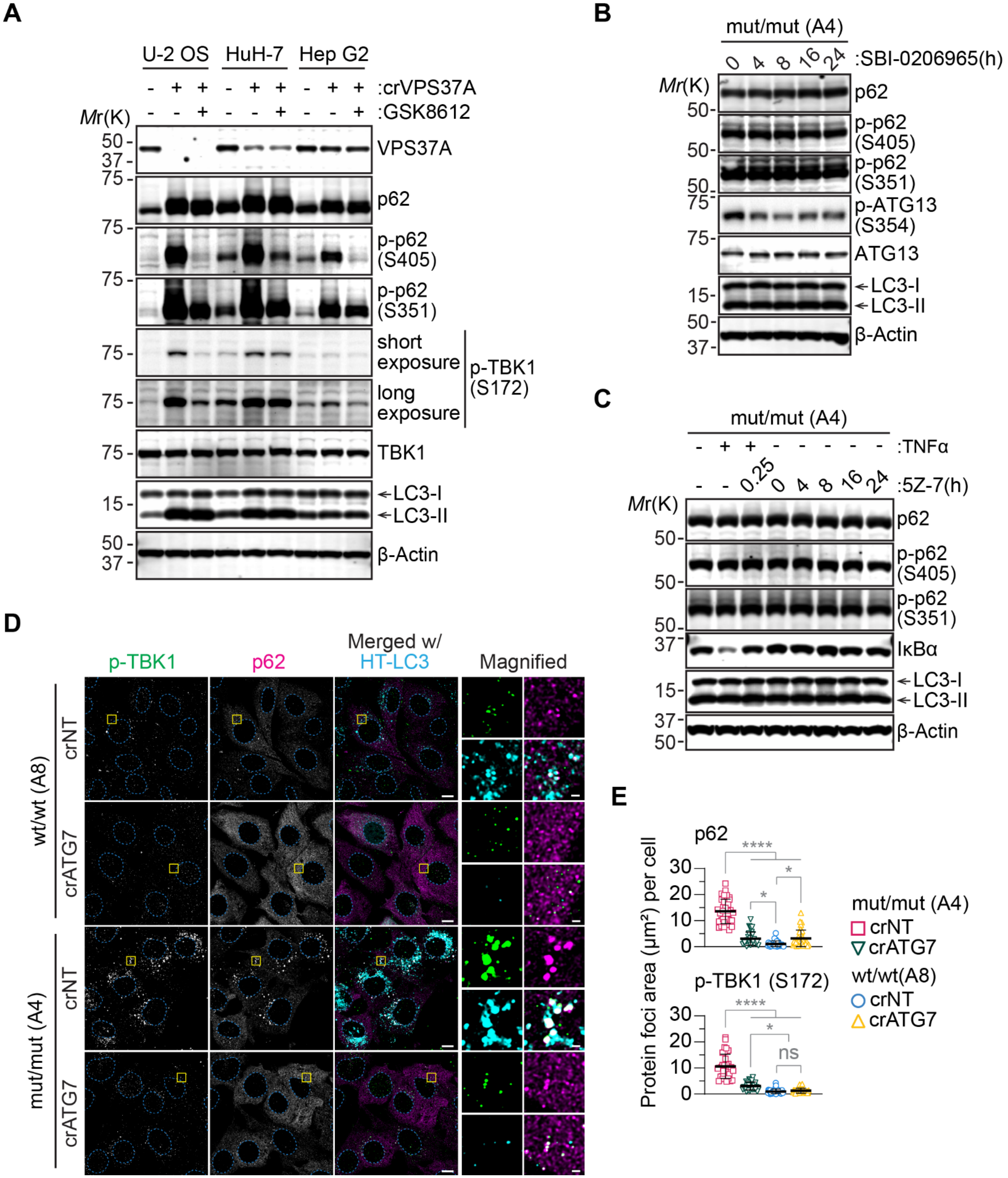

Figure S8. Inhibition of TBK1, but not TAK1 and ULK1, diminishes p62 phosphorylation and inclusion formation induced by VPS37A UEVL loss. (A) Immunoblot analysis of the indicated cells that were treated with 10 μM GSK8612 for 4 hours. (B and C) Immunoblot analysis of MEFs that were treated with 10 μM SBI-

0206965 (B) or 10 μM (5Z)-7-oxozeaenol (5Z-7) (C) for indicated periods of time. In (C), cells treated with 10 ng/mL TNFα in the presence or absence of 10 μM 5Z-7 for 15 min were used as controls for the detection of TAK1-dependent IκBα degradation. (D) Confocal images of HT-LC3-expressing MEFs that were incubated with MPL-TMR for 15 min and stained for p-TBK1 (Ser172) and p62. Scale bars: 10 μm; 1 μm in magnified images. (E) Bar plots of total p62 and p-TBK1 (Ser172) foci area (μm^2) in (D) (n = 35). Statistical significance was determined by one-way ANOVA followed by Tukey's multiple comparison test. All values in the graphs are mean ± SD. *$p \leq 0.01$; ****$p \leq 0.0001$; ns, not significant.
